# Antifluorite-type Na_5_FeO_4_ as a low-cost, environment-friendly cathode with combined cationic/anionic redox activity for sodium ion batteries: a first-principles investigation†

**DOI:** 10.1039/d2ra01834a

**Published:** 2022-06-13

**Authors:** Rasmus Vester Thøgersen, Federico Bianchini, Helmer Fjellvåg, Ponniah Vajeeston

**Affiliations:** Centre for Materials Science and Nanotechnology, Department of Chemistry, University of Oslo P.O. Box 1033 Blindern N-0315 Oslo Norway vajeeston.ponniah@smn.uio.no

## Abstract

The rapid electrification of our society and the transition towards a larger share of intermittent renewable energy sources in our electricity grids will dramatically increase the demand for cheap energy storage. Sodium ion batteries (SIBs) show a lot of promise to provide the required stationary storage at the grid level at low cost owing to the natural abundance and geographical availability of sodium. In addition, alkali-rich cathode materials exhibiting anionic redox contributions have garnered much attention over the past decade as a strategy to increase the specific capacity. In this work, we investigate for the first time the sodium-rich compound Na_5_FeO_4_ as a potential low-cost, environment-friendly cathode for sodium ion batteries from first principles using density functional theory. We investigate three low-energy polymorphs related to the antifluorite structure, verify their dynamical and mechanical stabilities, and show that they exhibit promising ion diffusive properties. As alkali-rich cathode materials are prone to oxygen loss during cycling, we investigate cycling stability with respect to phase transformations and oxygen loss and identify in particular one promising cycling interval that can reversibly shuttle 1.5 Na^+^ per formula unit between Na_5_FeO_4_ and Na_3.5_FeO_4_ with a gravimetric energy density exceeding 360 W h kg^−1^. Investigations into possible redox mechanisms reveal that the charge compensation occurs simultaneously on Fe- and O-atoms in FeO_4_-tetrahedra, which suggests that Na_5_FeO_4_, if realised experimentally as a cathode material, would join the family of combined cationic/anionic redox compounds.

## Abstract 2

Fukui-functions calculated for predicted phases upon desodiation of potential cathode material Na_5_FeO_4_, to Na_3.5_FeO_4_, showing predicted simultaneous redox activity associated with Fe and O electronic states.

## Graphical abstract

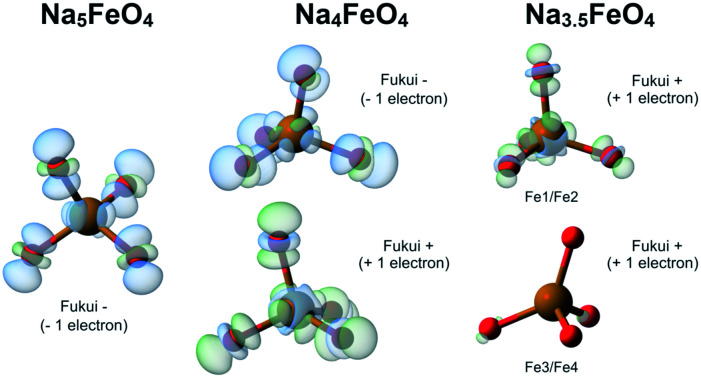

In the past decade, sodium ion batteries (SIBs) have attracted increasing attention as an alternative to lithium ion batteries (LIBs) for applications where the energy density is not of utmost concern, such as for stationary grid storage.^1^ For these applications, the levelised cost of energy (LCOE) is the most important factor, and this will in large depend on manufacturing costs and cycle lifetime. One way to lower the cost of the electrode materials is by only utilising earth abundant elements that are not prone to price volatility. SIBs are interesting in this respect due to the high abundance, geographical availability and correspondingly low cost of sodium compared to lithium. In addition, by only using iron as a transition metal redox centre, the total material cost of the cathode could indeed be very low. Furthermore, by employing SIBs for applications where the higher energy densities found in LIBs are not strictly required, regions without a natural abundance of lithium resources would be able to lessen their import dependence on critical raw materials (CRM) required for battery production, such as within the European Union where such a CRM independence is a stated goal.^2^ Continued concerns about lithium resource availability could also allow SIBs to play a larger role in the future energy system, alleviating pressure on lithium where it is not required and further contribute to CRM independence.^3–6^

Another topic that has generated a lot of interest in the academic community over the past decade is cathode materials that exhibit combined cationic and anionic redox activity.^7^ In these materials, as Li^+^/Na^+^ is removed from the compound during charging, there is a redox compensation not only from the oxidation of the transition metal, but also from a partial oxidation of an oxygen anion. This has been shown to arise from the existence of labile oxygen states close to the Fermi level.^8^ These alkali metal rich materials provide the essential environments surrounding the oxygen anions to enable the labile states, and at the same time substitutes heavier transition metal ions for the lighter alkali metal ions resulting in a higher theoretical specific capacity. Many of the researched materials have been layered structures, such as the proof-of-concept cathode Li_2_RuO_3_,^9^ and while most such materials have been prone to poor cycling stability due to an irreversible loss of oxygen from the lattice, advancements in our fundamental understanding of such materials suggest that the issues of structural instability at high deintercalation levels should be less pronounced in tridimensional structures.^8^

One such class of tridimensional materials are the antifluorite-type compounds, such as Li_5_FeO_4_, Li_6_CoO_4_ and Li_6_MnO_4_, which have all been studied as potential cathode materials for LIBs.^10,11^ Li_5_FeO_4_ has, for example, been shown to allow reversible extraction of up to 1.2 Li^+^, giving a specific capacity of just above 200 mA h g^−1^.^12^ The crystal structures of these compounds can all be described by considering a 2 × 2 × 2 supercell of the ideal antifluorite structure Li_2_O, with oxygen anions forming a cubic closest packed sublattice with lithium cations occupying all tetrahedral holes. Comparing Li_5_FeO_4_ with Li_2_O (Li_8_O_4_), three Li^+^ are replaced by one Fe^3+^ and two Li^+^-vacancies (*v*′_Li_). In Li_6_CoO_4_, two Li^+^ are replaced by one Co^2+^ and one *v*′_Li_. Their sodium analogues were reported to exist in the 70s and 80s by Hoppe and co-workers^13–16^ although with slightly different stoichiometries. In these cases, the sodium contents of the Co- and Mn-structures are lower, compensated by a higher oxidation state in the transition metal cations, and exhibiting other variants of the antifluorite structure. To our knowledge, there are no reports to date of these materials having been investigated for possible use as cathode materials for SIBs.

In this work, we investigate the sodium oxoferrate Na_5_FeO_4_ as a potential candidate as a cheap, environmentally friendly cathode for SIBs by use of density functional theory. After describing the computational details, we show results from an investigation of the phase stability based on experimentally known and theoretically predicted crystal structures of A_5_MO_4_ (A = alkali metals, M = transition metals) from the structure databases ICSD^17^ and Materials Project,^18^ and show how the relative stability of the most stable modifications shift with temperature. We verify that the low-energy modifications are all dynamically and mechanically stable, and verify that their diffusive properties are suitable for use as a cathode material based on migration barriers obtained from nudged elastic band-calculations (NEB). In order to predict how much Na^+^ can be expected to be reversibly extracted from the structure, we compare the phase stability of the desodiated phases with oxygen deficient structures and calculate the formation enthalpies for oxygen vacancies at different desodiation levels. Lastly, we discuss the possible redox mechanisms during desodiation from a structural and electronic point of view.

## 1 Computational details

Density functional theory (DFT) calculations are performed using the projected-augmented-wave (PAW)^19,20^ implementation of the Vienna *Ab initio* Simulation Package (VASP 5.4.4).^21–24^ The Perdew, Burke and Ernzerhof (PBE) functional is used for the exchange-correlation term.^25^ The Hubbard parameter *U* is used following the rotationally invariant form by Dudarev and Botton.^26^ The effective *U*-value for the d-orbitals is set to 5.3 eV for Fe in correspondance with calibrated values for oxide systems by the Materials Project, which were obtained by fitting to experimental binary formation enthalpies.^18^ The optimised structures are obtained by minimising the total energy with a convergence criterion of 10^−3^ eV using the conjugate gradient algorithm. The convergence threshold for the electronic self-consistent calculations are set to 10^−5^ eV. Brillouin zone (BZ) integration is performed with a *Γ*-centered grid with a smallest allowed spacing between *k*-points of 0.4 Å^−1^. The BZ integration is done according to the Tetrahedron method with Blöchl-corrections for construction of the energy–volume curves and electronic structure calculations.^27^ For subsequent calculations, Guassian smearing with broadening width of 0.2 eV was used. We explicitly include all 2p and 3s electrons for Na, all 2s and 2p electrons for O and all 3p, 3d and 4s electrons for Fe. The energy cutoff is set to 500 eV, and the calculations are converged to within 2.5 meV per atom with respect to a higher energy cutoff of 800 eV. The energy–volume curves are obtained by stepwise relaxation of the structure. In the first step, only the ionic positions are relaxed, in the second step both ionic positions and cell shape are relaxed, and finally both ionic positions, cell shape and cell volume are relaxed. From the relaxed structure we then construct fixed volume configurations by applying a diagonal strain in increments of 1% volume change in both the tensile and compressive directions, and allowing ionic positions to relax. The obtained energies are then fitted to the Birch–Murnaghan equation of state^28^ to obtain equilibrium values for the total energy, volume, bulk modulus and its pressure derivative.

The phonopy package is used to construct displacements for all phonon calculations.^29^ For these calculations, we construct supercells when needed to ensure that each lattice parameter is at least 10 Å. First, we allow the structure to relax using a stricter convergence criterion with a force convergence threshold of 10 meV Å^−1^ and electronic convergence criterion of 10^−8^ eV. The forces are then calculated for all displacements using the same electronic convergence criterion where all projection operators are evaluated in reciprocal space for added accuracy, as opposed to the automatic scheme provided by VASP used in all other calculations. We employ the finite differences method with displacements lengths of 0.1 Å. The phonopy package is further used to calculate the phonon dispersion relations, phonon density of states and thermal properties.

The single crystal elastic constants are calculated using the finite strain method, and the resulting symmetrised tensor is diagonalised using the NumPy package^30^ to obtain the eigenvalues.

Electronic density of states have been calculated by running a static, self-consistent calculation on a pre-relaxed structure with the smallest allowed spacing between *k*-points reduced to 0.1 Å^−1^ for a finer grid in *k*-space.

To assess the electrochemical behaviour, we calculate the formation energies Δ*E*_form_ of several Na-deficient compositions, Na_5−*x*_FeO_4−*y*_ (*x* = 0.0–3.0, *y* = 0.0–2.0), to construct the convex hull as outlined by Urban *et al.*^31^ from

1 Δ*E*_form_(1 − *X*) = *E*(1 − *X*) − (1 − *X*)*E*(1) − *XE*(0)

where the *E*(1) and *E*(0) are the total energies of the end compositions and *E*(1 − *X*) (0 ≤ *X* ≤ 1) is the total energy of an intermediate composition a distance *X* from *E*(0) and (1 − *X*) from *E*(1), from which *E*_form_(0) and *E*_form_(1) are set to 0 by construction. We used pymatgen to obtain the Na-deficient structures by generating all combinations of vacant structures and proceeding with the set of ten with the lowest unique Ewald sum for each of the deintercalation steps.^32^ In addition, we performed an extensive search, using matminer to search the Materials Project database^33^ and manually searching the ICSD-database, for structures matching the stoichiometry of the deintercalated structures, as well as for structures with a lower oxygen stoichiometry. The latter structures were included in order to assess when irreversible oxygen release is expected, and to compare these modifications with structures of higher oxygen content, we calculate their total energies as2
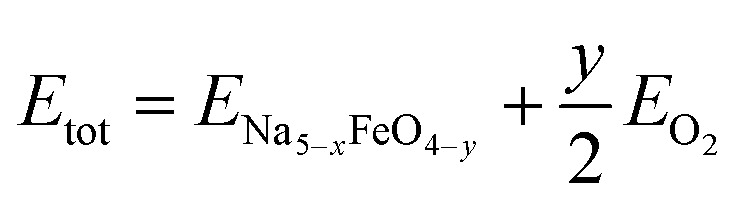


where *E*_Na_5−*x*_FeO_4−*y*__ is the ground state energy of the oxygen deficient modification and *E*_O_2__ is the total energy of a gas phase oxygen molecule.

We further treat the most stable desodiated structures along the convex hull and calculate the equilibrium voltages *vs.* Na^+^/Na(V) from the following equation:3
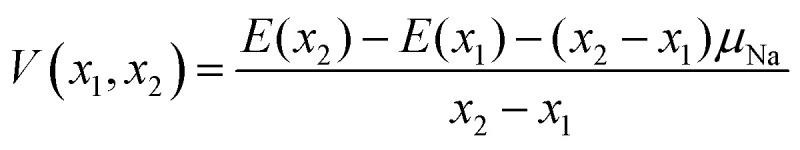


where (*x*_2_ − *x*_1_) is the number of deintercalated Na^+^-ions for the given voltage step and *E*(*x*_*i*_) is the total energy for the respective phase. The chemical potential *μ*_Na_ is set to the total energy per atom of the bcc form of Na metal.

The activation energies for the diffusion of Na^+^-ions are calculated using the NEB-method.^34^ First initial and final states of a given jump are created, before a set of five images are generated between these states from linear interpolation between the end points using VTST Tools.^34,35^ When needed, we create a supercell of minimum 10 Å × 10 Å × 10 Å to ensure sufficient spacing between the vacancy and its image in the neighbouring unit cells to avoid self-interaction in order to investigate diffusion in the dilute limit. We first conduct a NEB calculation to obtain an initial guess of the minimum energy path. Then the calculation is restarted using the climbing nudged elastic band (cNEB) method to obtain a more accurate result.^35^ The initial vacant structures are created by considering all Na–Na distances shorter than 3 Å, in certain cases extended somewhat to complete a percolating diffusion path. After removal of the appropriate Na-ions for each jump, the atomic positions are relaxed before creating the images. The ionic optimisation for the intermediate images are carried out using the FIRE algorithm^36^ with a force convergence threshold of 40 meV Å^−1^. The diffusion coefficients of Na^+^ (*D*_Na^+^_) as a function of temperature are estimated from

4 *D*_Na^+^_(*T*) = *d*^2^*ν*_0_exp(−*E*_a_/*k*_B_*T*)

where *d* is the hopping distance, *ν*_0_ is the attempt frequency (assumed to be 10 THz^37^), *E*_a_ is the activation barrier obtained by the NEB calculations and *k*_B_ is the Boltzmann constant.

The oxygen vacancy formation enthalpies are calculated based on the following reaction:5
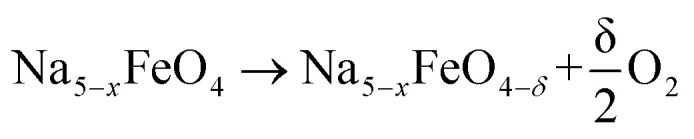


where 0 < *x* < 3 and *δ* is dependent on the size of the unit cell used, but in all cases limited to give a vacancy concentration below 3.13%. The vacancy formation energies Δ*E*_vac_ are calculated as6
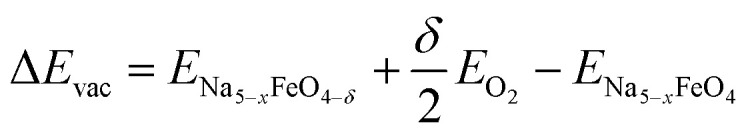


where *E*_Na_5−*x*_FeO_4__, *E*_Na_5−*x*_FeO_4−*δ*__ and *E*_O_2__ are the total energies of the pristine structures, the oxygen deficient structures and gas phase oxygen molecule, respectively. The oxygen deficient structures are generated in a similar manner as described above for the Na-deficient structures using pymatgen. To study the thermodynamic stability of the oxygen vacancies as a function of temperature, we substitute the total energy of the oxygen molecule with the temperature and pressure dependent chemical potential, given as7
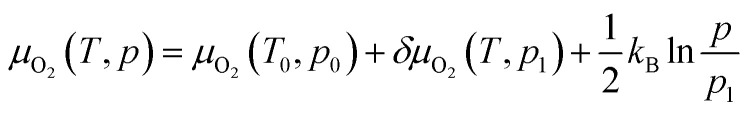


where *μ*_O_2__(*T*, *p*) is the chemical potential of molecular oxygen at temperatures *T* and pressure *p* and *μ*_O_2__(*T*_0_, *p*_0_) is the total energy obtained from DFT calculations (at *T* = 0 K and *p* = 0 bar). At a given pressure, *p*_1_, the temperature dependent contribution to the chemical potential is given as *δ*_O_2__(*T*, *p*_1_) and is obtained from the JANAF thermochemistry tables,^38^ and the last term is the pressure dependent contribution under the ideal gas assumption. Vibrational contributions to the entropy are not considered. The candidates for oxygen vacancies are calculated using the same method as for the desodiated phases, and only the atomic positions are allowed to relax in order to simulate vacancies in the dilute limit. To avoid self-interaction errors, the supercells are adjusted as needed to keep each lattice parameter above 10 Å.

The Atomic Simulation Environment (ASE) has been used for automisation of calculations and for data analysis.^39^ All crystal structures are rendered with VESTA.^40^

## 2 Structural stability

We performed a search for experimentally known and theoretically predicted compounds with the stoichiometry A_5_MO_4_ (A = alkali metals, M = p-, d- and f-block elements) in the ICSD and Materials Project databases to get a set of starting geometries for our investigation. The list of all starting geometries used are listed in Table S1 (ESI†).

**Fig. 1:**
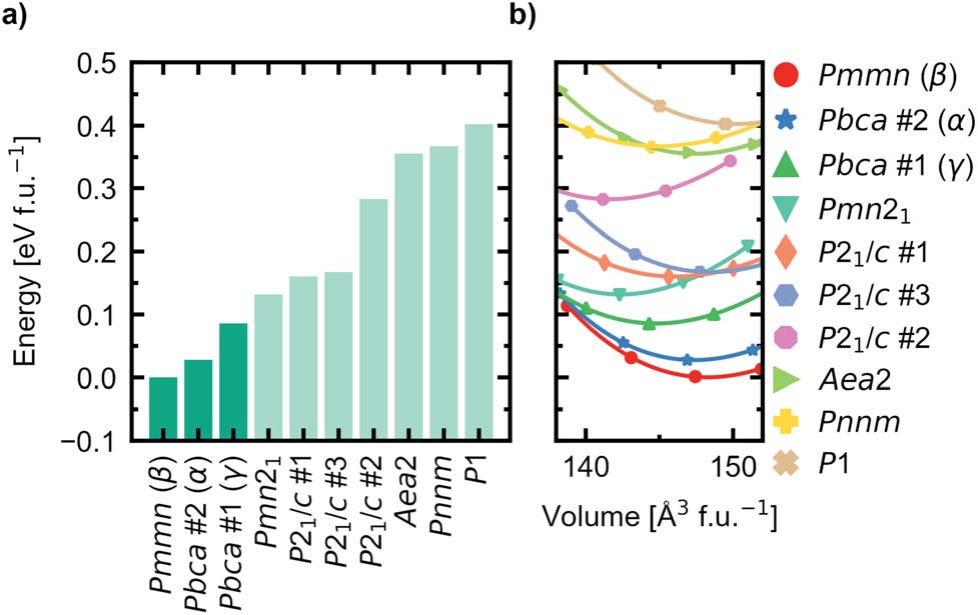
(a) Equilibrium energies of the ten lowest energy structures, with the three modifications chosen for further investigation highlighted in a darker colour. (b) Energy-volume curves fitted to the Birch–Murnagahn equation of state for the ten lowest energy trial structures.

For all starting geometries, the reference structures were subject to a simple ion substitution and volume change to obtain input structures with stoichiometry Na_5_FeO_4_ and the starting volume 147 Å^3^ f.u.^−1^. The volumes were changed preserving the ratio between the lattice axes. Energy-volume curves were computed for each of the starting geometries after being pre-relaxed as described in Section 1. The equilibrium energies, volumes, bulk moduli and its pressure derivative for all structures are summarised in Table S2.† The relative equilibrium energies along with the energy–volume curves of the ten structures with the lowest energies are shown in Fig. 1.

The most stable phase was found to have *Pmmn* space group symmetry (s.g. 59), with two distinct structures with *Pbca* space group symmetry (s.g. 61) lying within 100 meV f.u.^−1^ of the most stable phase. This energy difference was chosen as a cut-off for further investigations. In order of increasing energies, they are hereby designated as β-, α- and γ-Na_5_FeO_4_, respectively, the two former following the naming convention by Hoppe and co-workers^41,42^ and the latter given by the current authors. γ-Na_5_FeO_4_, which is predicted as the least stable of the three polymorphs at 0 K, corresponds to the experimentally reported structure of Na_5_FeO_4_ reported by Brachtel and Hoppe in 1978 (ref. 13) and by Fruchart *et al.* in 1985,^43^ and is shown later in this work to stabilise at higher temperatures when also including the entropic contributions. The α-phase corresponds to the experimentally reported structure of Li_5_FeO_4_ by Luge and Hoppe in 1984,^44^ and the β-phase corresponds to the reported structures of Na_5_InO_4_ and Li_5_AlO_4_, also reported by Hoppe and co-workers.^41,45^

**Fig. 2:**
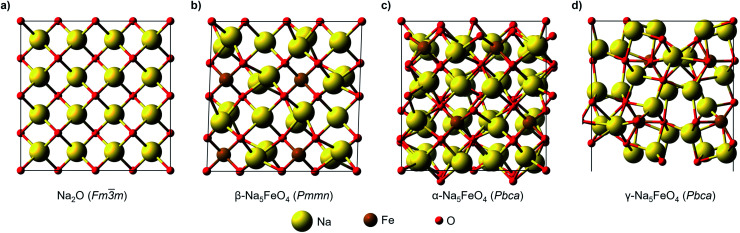
(a) A 2 × 2 × 2 supercell of Na_2_O (*Fm*3̄*m*). (b) Supercell of the β-polymorph (*Pmmn*) that has undergone a unit cell transformation with new lattice vectors 
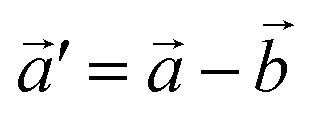
, 
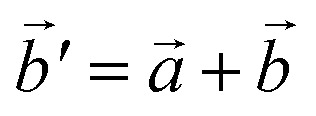
 and 
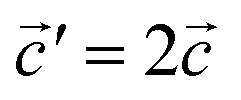
. (c) Unit cell of the *α*-polymorph (*Pbca*). (d) Partial view of a supercell of the *γ*-polymorph (*Pbca*), with 
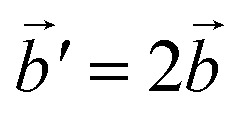
 and cut in half along the [001]-direction to include the eight face-centered cubes of the distorted O^2−^-sublattice.

The structural data for these three polymorphs are summarised in Table S4,† and the primitive unit cells are shown in Fig. S1.† All of these three phases are related to the antifluorite structure, and can be described as defect and distorted variants of the ideal structure, as shown in Fig. 2. The primitive unit cell of the α-phase corresponds to a 2 × 2 × 2 supercell of the antifluorite structure, while the primitive unit cell of β-Na_5_FeO_4_ can be transformed to arrive at the same antifluorite-type supercell, by employing a new set of lattice vectors: 
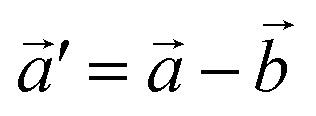
, 
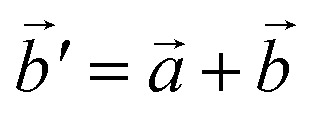
 and 
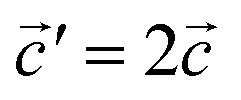
. The γ-phase can be thought of as a distorted 2 × 1 × 4 supercell of the ideal antifluorite structure, where only the top half of the supercell is shown in Fig. 2d. The antifluorite structure, exemplified by Na_2_O (*Fm*3̄*m*, s.g. 225), has a cubic closest packing of O^2−^, with Na^+^ in all tetrahedral holes, as seen in Fig. 2a. In Na_5_FeO_4_, Fe^3+^ replaces one Na^+^ in each of the octants and is charge compensated by the creation of two Na^+^-vacancies (*v*′_Na_). The three structures differ from each other in the ordering of Fe^3+^ and *v*′_Na_ relative to the other octants, and the degree of deviation of the oxygen sublattice from the ideal cubic closest packing. The β-phase deviates the least from this ideal packing and retains a readily identifiable packing sequence. So does the α-phase, but here the oxygen sublattice is further distorted. The γ-phase deviates the strongest from the ideal antifluorite structure with alternating depressions in the cubic octants, highlighted in Fig. S2,† making it hard to identify the relation to the antifluorite structure at first glance.

The ordering of Fe^3+^ in both the β- and γ-polymorphs is such that entire sheets of only Na^+^ can be identified, where Na^+^ can be thought to diffuse more freely, whereas in the α-phase, no such sheets can be isolated. In the case of the β-phase, these sheets are found parallel to the (100)- and (010)-planes, and in the γ-phase such sheets exist parellel to the (100)-planes.

In the ideal cubic antifluorite structure, all O–O-distances are the same, but as Fe^3+^-cations and Na^+^-vacancies are introduced and the oxygen sublattice distorted, these distances will start to differ depending on the relative proximity of each O^2−^ to Fe^3+^, Na^+^ or *v*′_Na_, respectively. In the Li-analogue, Li_5_FeO_4_, all FeO_4_-tetrahedra are still connected to neighbouring LiO_4_-tetrahedra or *v*′_Li_O4-tetrahedra, meaning that there are shorter O–O-distances connecting these tetrahedra than some of the internal O–O-distances within the FeO_4_-tetrahedron itself. In Na_5_FeO_4_, however, the FeO_4_-tetrahedra become completely isolated. For this reason, this structure type is also referred to as an island structure by Hoppe^13^ and denoted Na_5_[FeO_4_]. This is the case for all polymorphs considered here.

**Fig. 3:**
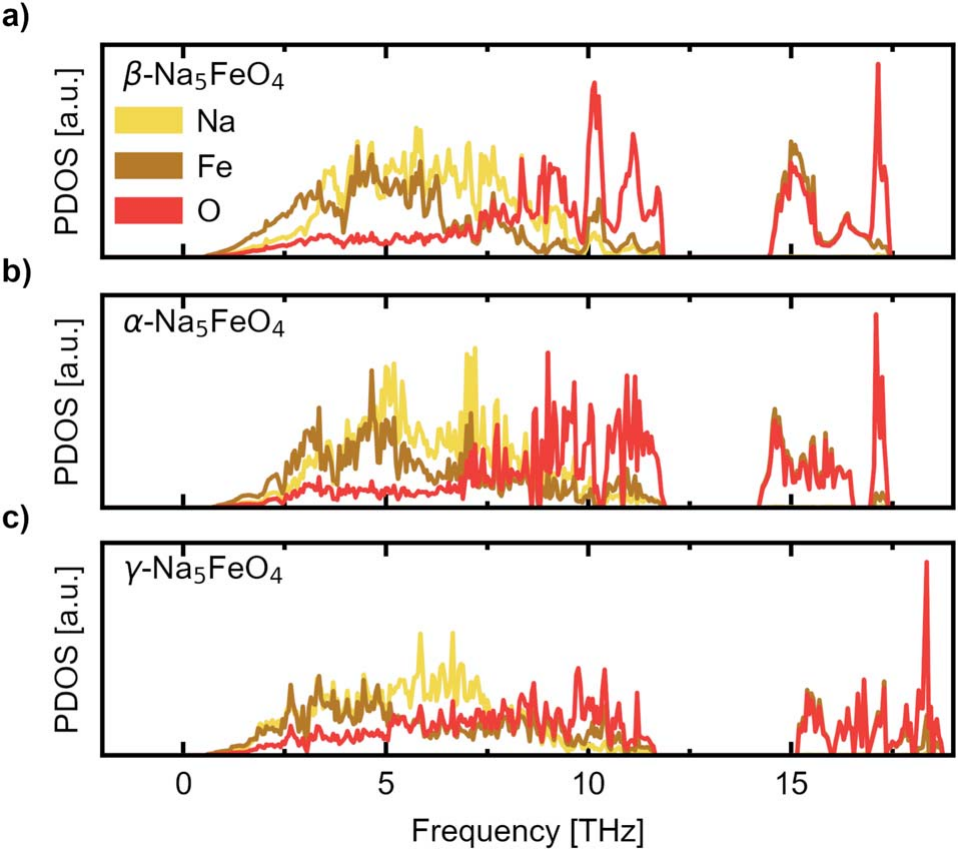
The site-projected phonon density of states (PDOS) normalised per atom for (a) the β-polymorph, (b) the α-polymorph and (c) the γ-polymorph.

We further investigate the mechanical and dynamical stability of each of the polymorphs. First, in order to assess the mechanical stability, we computed the single crystal elastic constants. The resulting matrices were diagonalised and resulted in only positive valued eigenvalues, indicating that all considered polymorphs are mechanically stable. The eigenvalues are given in Table 1 while the full symmetrised elastic moduli are given in Table S3.† Phonon calculations were then carried out in order to assess the dynamical stability of each phase, and to investigate any changes in relative stability with temperature. The atom-projected phonon densities of states (PDOS) are given in Fig. 3, while the full dispersion relations are given in Fig. S3–S5.† As can be seen, there is an absence of any imaginary phonon modes in all polymorphs, indicating that they are all predicted to be dynamically stable. As the crystal structure of β-Na_5_FeO_4_ is less distorted than for the other phases, this phase is also expected to have more degenerate vibrational modes and thus more defined peaks, which is also clearly visible. In all cases, Fe and Na dominate at lower frequencies, but while contributions from Fe diminishes from 5–6 THz and upwards, the Na-contributions remain considerable for several THz above this. In this region in particular, the independence of Na from the other atoms is visible, indicative of decent diffusive properties, which are investigated further with NEB-calculations in Section 3. Above the phononic band gap there are many degenerate states composed of Fe and O, indicating stiff bonds of more covalent nature.

**Table 1:** Eigenvalues (GPa) resulting from the diagonalisation of the elastic moduli, showing only positive values indicating mechanical stability of all three phases

Phase	1	2	3	4	5	6
β-NFO	161.9	52.1	67.1	19.3	33.9	14.9
α-NFO	158.1	39.3	50.3	30.8	31.6	27.9
γ-NFO	144.6	44.7	59.4	27.6	28.7	26.1

**Fig. 4:**
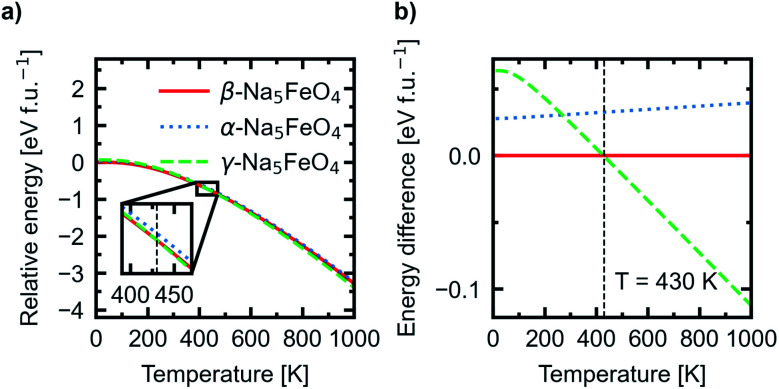
(a) The temperature adjusted relative energies of the α-, β- and γ-polymorphs. Inset shows the crossover point where γ-Na_5_FeO_4_ becomes the most stable polymorph. (b) The difference in total energies as a function of temperature with the total energy of the β-phase set as the reference.

In addition, the Helmholtz free energies were calculated from the phonon calculations as a function of temperature, given by *F*(*T*) = *E*_DFT_ + *E*_vib_ − *TS*, where *E*_DFT_ is the ground-state energy obtained through conventional energy minimisation and *E*_vib_ − *TS* are the vibrational contributions to the Helmholtz free energy. The results are shown in Fig. 4. We see that the experimentally reported γ-phase, although disfavoured at 0 K, has a larger energy gain with temperature and becomes stabilised with respect to the other polymorphs. Our calculations predict that γ-Na_5_FeO_4_ becomes the most stable polymorph at around 430 K, as can be seen in the inset of Fig. 4a and in Fig. 4b. From Fig. 1b, it can be seen that also at lower volumes (*i.e.* higher pressures), there is a shift in relative stabilities, where both the β- and α-phases are destabilised with respect to the γ-phase.

This compound has previously been synthesised *via* a high-temperature solid state reaction route,^13,43^ but since these structures are so close in total energy, it could mean that the other polymorphs would be available through careful tuning of the synthesis parameters, as a function of particle size or through slight chemical modifications.

## 3 Ionic transport

The diffusivity of Na^+^-ions in the three most stable phases through vacancy diffusion was further calculated using the NEB-method as detailed in Section 1. Jumps to interstitial sites are for practical reasons not considered here. All atomic labels herein refer to the Wyckoff positions listed in Table S4,† and the directions refer to the antifluorite-type unit cells described in Section 2. A summary of the diffusion paths identified, as well as a summary of each individual jump, can be found in Tables S5 and S6.†

**Fig. 5:**
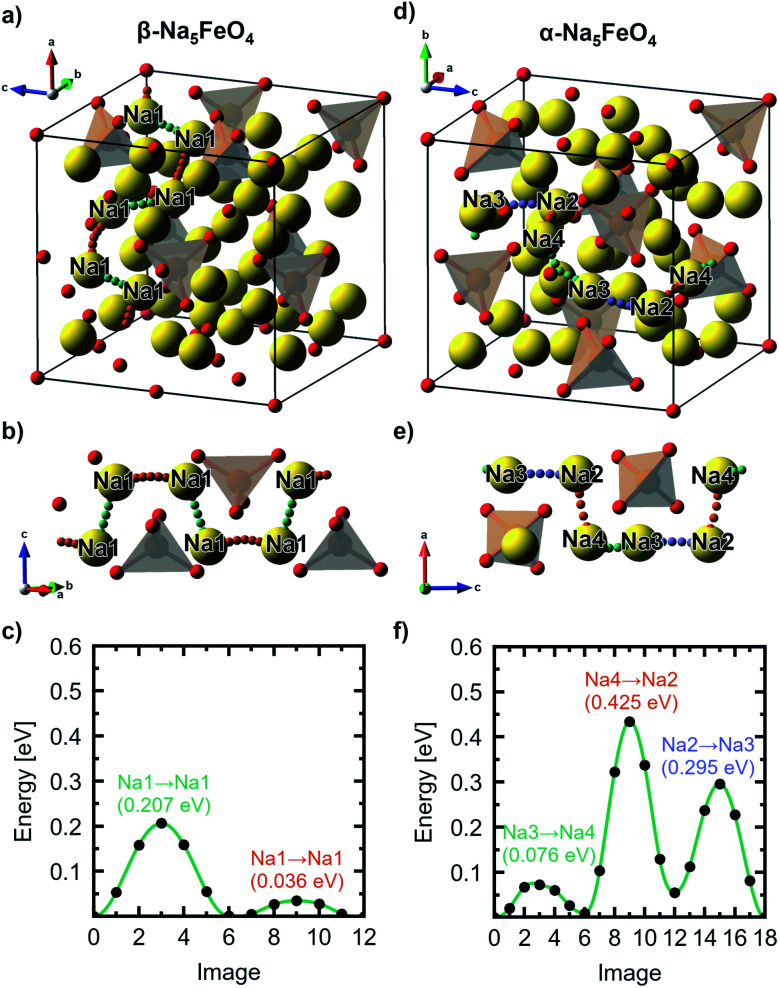
(a) The most facile diffusion path through the antifluorite-type unit cell, (b) a cutout of the diffusion path and (c) the associated migration barriers calculated from NEB-calculations, respectively, of the β-polymorph. (d–f) shows the same for the α-polymorph. All labels refer to the Wyckoff site occupation tabulated in Table S4.†

For the β-phase, four unique Na–Na-jumps were identified giving rise to five unique diffusion pathways, two having a net movement in the [110]-direction, one along [001] and two with a net movement along [11̄0]. The most facile path has an overall barrier of 0.207 eV with a net movement in the [110]-direction involving two distinct jumps between Na1. Based on the limiting energy barrier along this path (0.207 eV), and the Na–Na-distance associated with this jump (2.79 Å), the diffusion coefficient along this direction is calculated according to eqn (4) to be 2.66 × 10^−6^ cm^2^ s^−1^ at 300 K, being by far the highest diffusion coefficient of all the polymorphs. The diffusion pathway and associated energy barriers are shown in Fig. 5a–c. The movement is contained within a sheet free of any Fe^3+^-ions that exist in this phase due to the particular ordering of the transition metal cations. The remaining paths have barriers that range from 0.301–0.406 eV. Of these, two are also purely contained within sheets of Na, while the two others have a more tortuous path that involves movement between planes.

For the α-polymorph, ten unique jumps were identified which give rise to four percolating pathways, with two having a net movement in the [001]-direction, one along [010] and the last along [100]. Here, the lowest overall barrier was calculated to be 0.434 eV along [001], following the pathway Na2–Na4–Na3, shown in Fig. 5d–f. The diffusion coefficient is calculated to be 1.4 × 10^−9^ cm^2^ s^−1^ based on the limiting jump along this pathway, between Na2 and Na4, with a jump distance of 2.88 Å and an energy barrier of 0.425 eV. This is, notably, three orders of magnitude lower than what was found in the β-phase. The diffusion path along the [010]-direction, and the other path along [001], are limited by the same jump, and have calculated diffusion coefficients of 1.08 × 10^−10^ cm^2^ s^−1^. The last diffusion path along [100] involved a jump that is not well-described as a jump between two Na-sites, and as diffusion *via* interstitials and/or interstitialcies is not treated here, the reported values are based on the jump with the highest barrier under the assumption that this truly is the limiting jump along the diffusion path.

For the γ-phase, four unique jumps were identified giving rise to two unique pathways along the [100]- and [001]-directions, respectively. However, none of these pathways consisted purely of jumps from one Na-site to another, which indicates that diffusion through interstitials and/or interstitialcies must be considered for an accurate description of diffusive properties of γ-Na_5_FeO_4_. However, along both pathways, there is a relatively large barrier (0.364 eV) that is assumed to be the limiting jump in order to give an estimation of the diffusive properties in this polymorph.

In Fig. S6† the diffusion coefficients for the most facile diffusion paths for each polymorph are shown as a function of temperature as calculated from eqn (4). The values of the diffusion coefficients are quite high compared to experimental values reported for other known SIB cathode materials.^46–49^ If the values predicted in this work could be achieved experimentally, especially if the β-phase could be realised, Na_5_FeO_4_ could show excellent rate capabilities suitable for high power applications.

## 4 Structural stability upon desodiation

In order to assess a likely path for stable electrochemical cycling, we compared desodiated structures based on the three pristine polymorphs with other structure types obtained through a similar search as in Section 2 for Na_5−*x*_FeO_4_ (*x* = 0.0, 0.5, 1.0, 1.5, 2.0, 2.5, 3.0). As the number of desodiated configurations scales with the binomial distribution as *x* increases, with a peak at *x* = 2.5, only a subset of all possible configurations was considered as outlined in Section 1. Since O_2_-outgassing might be expected in Na-rich cathode materials during charging, we also considered oxygen deficient structures given by Na_5−*x*_FeO_4−*y*_ (*y* = 0.5, 1.0, 1.5, 2.0). These structure types were also obtained through a similar structure search as above. We refer to desodiated variants derived from the pristine phases by their pristine label, while all other structure types are grouped together based on oxygen stoichiometry.

**Fig. 6:**
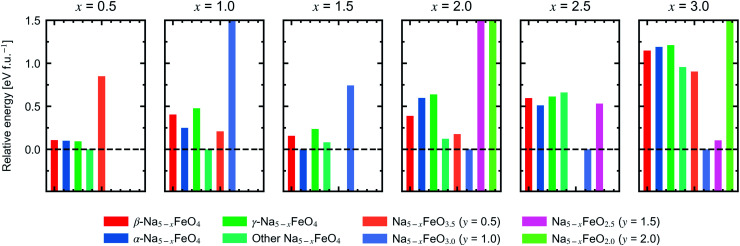
Relative energies of Na_5−*x*_FeO_4−*y*_ (*x* = 0.5–3.0, *y* = 0.5–2.0). Each panel corresponds to a given level of desodiation, and each bar corresponds to the lowest energy modification within its respective group of trial structures. Three of the groups are desodiated variants of the β-, α- and γ-polymorphs, respectively, while the rest are made up of structures from a database search for the given stoichiometry. Where there are no bars, no structure matching the stoichiometry showed up in the search. For each level of desodiation, the energies are given relative to the lowest energy modification.

For each level of desodiation, *x*, we compare the total energies between these groups to evaluate the reversibility of desodiation, where the total energies of the oxygen deficient modifications are calculated from eqn (2). We considered a desodiation step irreversible once oxygen deficient structures are preferred. The modifications with the lowest total energies from each group are plotted in Fig. 6, and it can be seen that up to 1.5 Na^+^ can be extracted before phases with lower oxygen content are favoured. Based on this, we found that there are two intervals of stable cycling without associated oxygen loss: (1) Na_5_FeO_4_ ⇌ Na_3.5_FeO_4_ + 1.5Na^+^ and (2) Na_3.5_FeO_3_ ⇌ Na_2_FeO_3_ + 1.5Na^+^. To access the second interval, there must necessarily be a formation step with initial oxygen loss to arrive at Na_3.5_FeO_3_ before the subsequent cycling can occur without further loss of oxygen. Such a cycling window could be of interest, where the initial cycling window could serve as a presodiation step, although the associated O_2_-evolution might pose safety hazards.

**Fig. 7:**
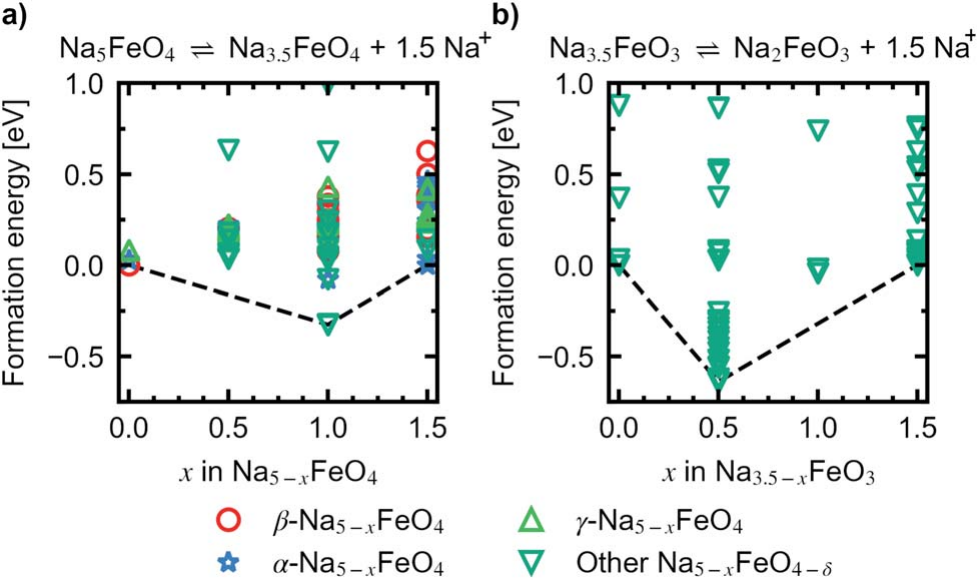
Convex hull constructred for the reactions (a) Na_5_FeO_4_ ⇌ Na_3.5_FeO_4_ + 1.5Na^+^ and (b) Na_3.5_FeO_3_ ⇌ Na_2_FeO_3_ + 1.5Na^+^.

**Fig. 8:**
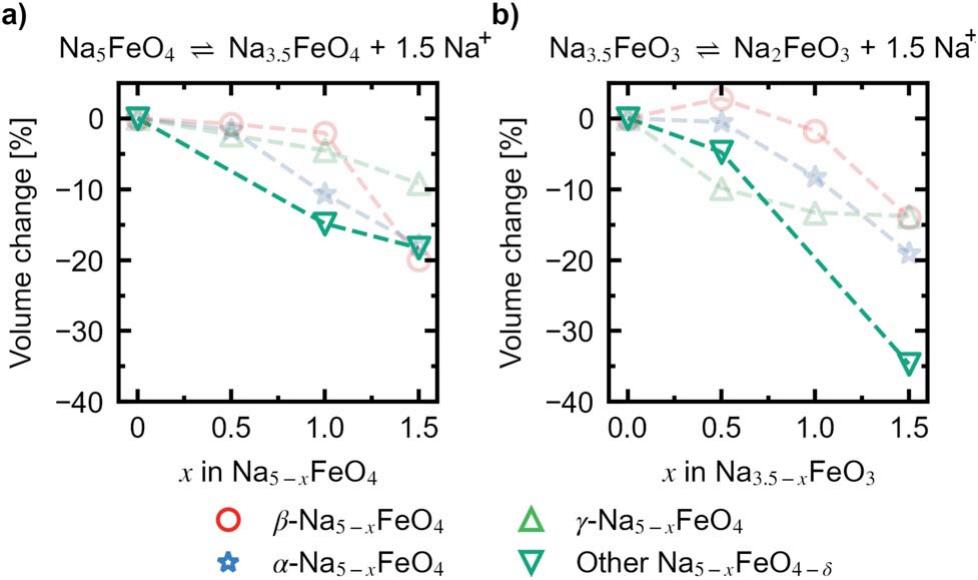
Relative volume changes corresponding to the reactions (a) Na_5_FeO_4_ ⇌ Na_3.5_FeO_4_ + 1.5Na^+^ and (b) Na_3.5_FeO_3_ ⇌ Na_2_FeO_3_ + 1.5Na^+^. Volume changes of desodiated variants of the β-, α- and γ-polymorphs are plotted for comparison in faded colours. The volume changes are given with respect to the initial volume for the given series.

**Fig. 9:**
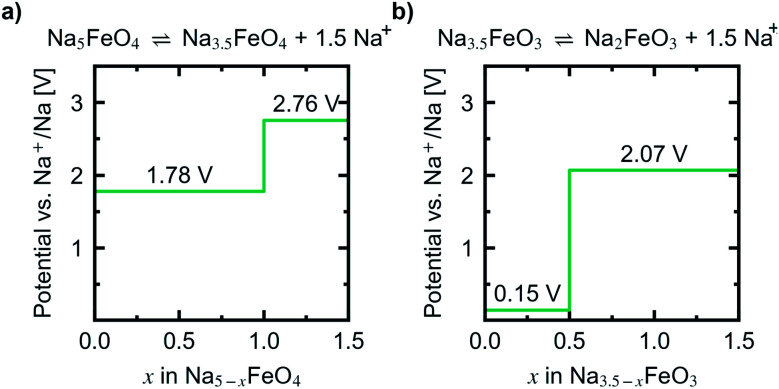
Predicted potential profiles *vs.* Na^+^/Na for the reactions (a) Na_5_FeO_4_ ⇌ Na_3.5_FeO_4_ + 1.5Na^+^ and (b) Na_3.5_FeO_3_ ⇌ Na_2_FeO_3_ + 1.5Na^+^.

Convex hulls were constructed for these two intervals according to eqn (1), and are shown in Fig. 7. For the first interval we identified two plateaus corresponding to Na_5_FeO_4_ → Na_4_FeO_4_ + 1Na^+^ and Na_4_FeO_4_ → Na_3.5_FeO_4_ + 0.5Na^+^. The associated structures of Na_4_FeO_4_ and Na_3.5_FeO_4_ have triclinic (*P*1̄, s.g. 2) and monoclinic (*P*2_1_, s.g. 4) space group symmetries, respectively, and structural data for these structures is tabulated in Table S7.† Both phases are still related to the pristine phase, preservering the isolated FeO_4_-tetrahedra, and retaining the general packing of the oxygen sublattice, although with further distortions. This indicates that this cycling interval should not be prohibited by excessive structural rearrangements. The associated volume changes along this desodiation path is shown in Fig. 8a, showing a volume contraction of just under 18%. This contraction is on the same order of magnitude as the volume expansion in the common SIB anode material hard carbon,^50^ providing a good match to reduce the overall stress associated with these volume changes in the cell during cycling. The predicted equilibrium voltages for these plateus were calculated using the phases along the convex hull according to eqn (3), and found to be 1.78 V for the first plateu and 2.76 V for the second, corresponding to an average voltage of 2.11 V for the whole interval, shown in Fig. 9a. With the exchange of 1.5 e^−^, this gives a specific capacity of 171.23 mA h g^−1^ and a gravimetric energy density of 361.30 W h kg^−1^.

For the second interval, a convex hull was similarily constructed (Fig. 7b) including only oxygen deficient phases, to reveal another two plateaus: Na_3.5_FeO_3_ → Na_3_FeO_3_ + 0.5Na^+^ and Na_3_FeO_3_ → Na_2_FeO^3^ + 1Na^+^. The corresponding potential profile (Fig. 9b) exhibits a very low voltage of 0.15 V for the first plateu and 2.07 V for the second. The very low voltage of the first step limits the practical use to the interval Na_3_FeO_3_ ⇌ Na_2_FeO_3_ + 1Na^+^. This corresponds to a specific capacity of 114.15 mA h g^−1^ and an energy density of 236.29 W h kg^−1^ for the limited cycling window. In this case, the initial phase (Na_3.5_FeO_3_) has triclinic (*P*1, s.g. 1) space group symmetry, while the desodiated phases (Na_3_FeO_3_ and Na_2_FeO_3_) both have monoclinic (*P*2_1_/*c*, s.g. 14 and *C*2/*m*, s.g. 12) space group symmetries, for which structural data is tabulated in Table S8.† These structures differ considerably from each other, and the Fe-coordination changes from edge-sharing tetrahedra, through corner-sharing tetrahedra, to edge-sharing octahedra in the last stage. This would require quite large reconstructions between each de-/sodiation step and is likely kinetically prohibitive. In addition, the volume changes associated with this cycling interval (Fig. 8b) are much larger than for the first interval. For these reasons, this cycling interval is not further investigated.

To look further into the stability of desodiation with respect to oxygen loss, we calculated the oxygen vacancy formation enthalpies for different levels of desodiation along the convex hull and for each of the three phases, according to the method outlined in Section 1. The most stable configurations of the desodiated variants previously obtained were used as the basis for these calculations. The vacancy concentrations for each considered modification, along with the vacancy formation enthalpies at 0 K, are summarised in Table S9.†

**Fig. 10:**
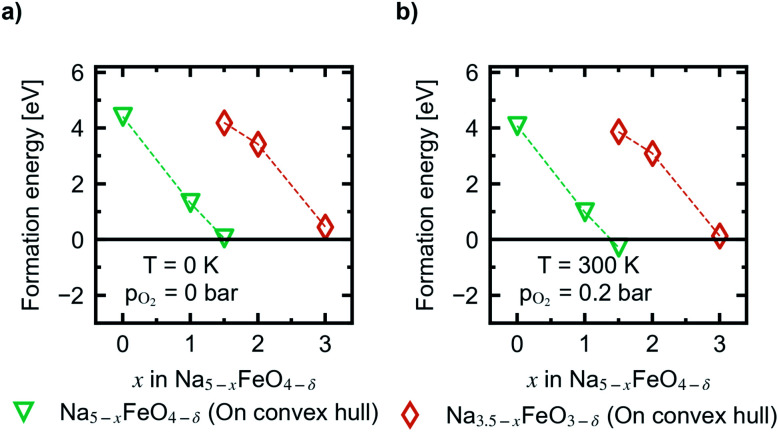
The evolution of vacancy formation energies as a function of *x* in Na_5−*x*_FeO_4_ at (a) *p*_O_2__ = 0 bar and *T* = 0 K, and (b) *p*_O_2__ = 0.2 bar and *T* = 300 K.

In Fig. 10 the evolution of the vacancy formation energies as a function of desodiation level *x* for the phases found along the convex hull is given for *p*_O_2__ = 0 bar at *T* = 0 K (Fig. 10a) and adjusted for ambient conditions, *p*_O_2__ = 0.2 bar and *T* = 300 K (Fig. 10b). The evolution of the vacancy formation energy with temperature is given for each individual composition in Fig. S7.† Oxygen vacancies are heavily unfavoured in the fully discharged state. However, as the structures are desodiated, the vacancies are quickly stabilised. Along the convex hull, the formation of an oxygen vacancy is barely unfavoured at 0 K at *x* = 1.5 (0.05 eV), however adjusting for temperature and pressure, the vacancy becomes slightly favoured at this level of desodiation (−0.28 eV). This is consistent with what is shown in Fig. 6, where we saw that oxygen deficient phases become stable after this level, and these results show that we can begin to expect a small amount of irreversible oxygen loss already at *x* = 1.5. As the vacancy concentrations are quite low, this should not be prohibitive, as any further oxygen loss at this point is found to be unfavourable. Furthermore, this decrease in vacancy formation energies with desodiation is also an indication that a certain amount of charge compensation can be attributed to oxygen, and that there is reversible contribution of anionic redox up until a point where oxide ions are fully oxidised and irreversibly disassociates from the lattice. The possible charge compensation mechanism will be expanded upon in the next section.

As expected, for the second cycling plateau, for which the compound has already suffered considerable oxygen loss, further vacancy formation is again heavily unfavoured at the start of the cycling interval. It also trends towards stabilisation upon further desodiation, however, it is never predicted to become stable at ambient conditions within the cycling interval.

## 5 Electronic structure and charge compensation

**Fig. 11:**
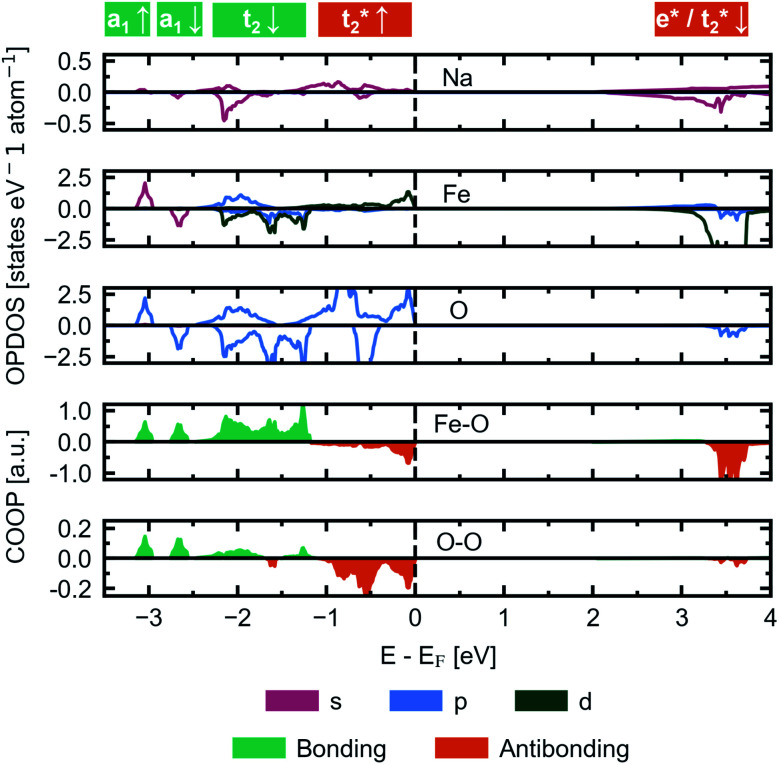
The site and orbital projected electronic density of states (OPDOS) normalised per atom for Na, Fe and O, respectively, along with the crystal orbital overlap population (COOP) computed for Fe–O and O–O interactions within the FeO_4_-tetrahedra in β-Na_5_FeO_4_. The spin up channel is plotted with positive values and the spin down channel is plotted with negative values in the OPDOS-plots. Bonding interactions are plotted with positive values and antibonding interactions are plotted with negative values in the COOP-plots. The Fermi-level (*E*_F_) is by convention set to 0 and is indicated by a vertical, dashed black line.

We herein report on the electronic structure of the pristine compound and the phases along the convex hull in the cycling interval Na_5_FeO_4_ → Na_4_FeO_4_ + 1Na^+^, and further discuss the charge compensation mechanism upon desodiation in this interval. In Fig. 11, the site and orbital projected electronic density of states (OPDOS) is given for each species for the pristine compound Na_5_FeO_4_, along with the crystal orbital overlap population (COOP) of the Fe–O and O–O interactions within the FeO_4_-tetrahedra. By convention, the Fermi-level (*E*_F_) is set to 0 eV. The expected features from a molecular orbital approach of a tetrahedrally coordinated Fe^3+^ compound with a high on-site repulsion of the d^5^-configuration are reproduced. Below *E*_F_, the *t*_2_-and *t*_2_*-states show a mixed Fe/O-character, although the O-states dominate, making this a charge transfer band gap. The band gap is computed to be just below 2 eV, however the number of available states at the bottom of the conduction band is quite small, and the majority of available states are found in the empty Fe 3d-states at about 3.5 eV above *E*_F_. This can be more clearly seen in the full bandstructure, given in Fig. S8.† This should be non-prohibitive from an electrochemical perspective, as shown by the commercial success of LiFePO_4_ that has a band gap exceeding this,^51^ where the combination of conductive additives and particle size reductions are used as means to facilitate electron transport to the redox centres,^52^ and a similar strategy could be pursued for Na_5_FeO_4_.

The low contribution of Na states around *E*_F_ confirms the ionic nature of the Na–O interactions, while the high degree of degeneracy of Fe 3d- and O 2p-states indicate a rather large degree of covalency in the Fe–O interactions, as previously seen in the phononic density of states. As is the case in other Li/Na-rich transition metal oxides, the environments surrounding each oxygen lead to localised O 2p states, often referred to as O 2p “lone pairs”. The oxide ions in Na_5_FeO_4_ are not cubically coordinated as in the ideal antifluorite structure, but instead octahedrally coordinated, with the octahedra being heavily distorted. In these octahedra, due to the presence of Na–O–Na bridges, O 2p-orbitals pointing along these directions are left essentially unhybridised as lone pairs on the oxide ions. The presence of these lone pairs can be identified through the computation of the electron localisation function (ELF), a topological tool of the electron density that identifies regions in space where paired electrons are localised.^53^ Indeed, electron pairs can be shown to exist along these directions as shown in Fig. S13a.† These localised oxygen states are also evident in the OPDOS, where one pair can be found as sharp peaks in both spin channels at around 1 eV below *E*_F_ and the other at around 2 eV below, although here not as readily identifiable, as these are not quite as localised and overlaps the *t*_2_-states. Due to the rather large distortion of the structure, there is bound to be a certain degree of overlap that would otherwise be symmetry disallowed, and this could lead to a lower degree of localisation for the lone pair states at lower energies. Interestingly, these lone pairs that are otherwise widely accepted to be the origin of anionic redox in other Li/Na-rich compounds,^8^ seems to be inactive upon desodiation, as seen in Fig. S13b and c.† The localised electron pairs are still present even at *x* = 1.5, with no apparent change at the same isosurface values.

**Fig. 12:**
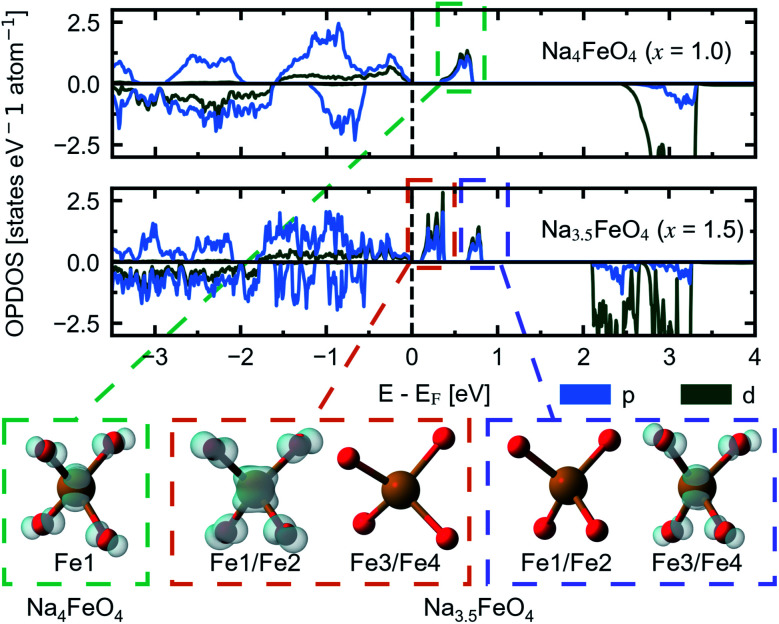
The site and orbital projected electronic density of states (OPDOS) for O 2p and Fe 3d for Na_4_FeO_4_ (*x* = 1.0) and Na_3.5_FeO_4_ (*x* = 1.5). Insets show the partial charge densities of the symmetry equivalent FeO_4_-tetrahedra associated with the “hole” states that appear upon desodiation. The labels of Fe corresponds to Wyckoff positions listed in Table S4.† For Na_3.5_FeO_4_, Fe1/Fe2 and Fe3/Fe4 are pairwise almost identical, and treated as two distinct Fe-sites.

This perserverance of the highly localised O 2p-states are also evident in the OPDOS of the desodiated phases, as seen in Fig. 12, however, as the structure becomes more distorted, the peaks become less sharply defined. Instead, “hole” states composed of a combination of empty Fe 3d- and O 2p-states become evident above *E*_F_, first one at *x* = 1.0 and then a second one at *x* = 1.5. The Fe 3d-states make up about a quarter of the total number of states in each of these holes. Upon integration of the hole states at *x* = 1.0, we find that the hole corresponds to a single electron per formula unit, as expected. For *x* = 1.5, the hole closest to *E*_F_ integrates to one electron per formula unit and the hole at a slightly higher energy level sums up to half an electron per formula unit. In such a situation we would expect a charge disproportionation where half the redox centres will have donated two electrons for charge compensation, while the other half will have donated only one. This is also what is observed from visualisation of the electronic states associated with each hole at different FeO_4_-tetrahedra, as seen in the inset of Fig. 12. This is also evident from inspection of the structural changes, shown in Fig. S14:† for *x* = 0.0 and 1.0 there is only a single distinct Fe-site, while for *x* = 1.5 there are four. Among those four, two and two and pairwise nearly identical and are subsequently labelled Fe1/Fe2 and Fe3/Fe4. One of these pairs is very similar to the FeO_4_-tetrahedron found in *x* = 1.0, while the other is further changed. The Fe–O distances are lowered for each desodiation step, from 1.93–1.94 Å at *x* = 0.0, to 1.81–1.83 Å at *x* = 1.0, to 1.73–1.77 Å for the most oxidised tetrahedra at *x* = 1.5. These values match well with what is expected from using ionic radii of Shannon^54^ of Fe with oxidation states +3, +4 and +5 (obtained by interpolating values for tetrahedrally coordinated Fe between Fe^3+^ and Fe^6+^): 1.89 Å, 1.81 Å and 1.73 Å. The distances between oxygen ions within each tetrahedron also decreases with desodiation, and this is further discussed below.

**Fig. 13:**
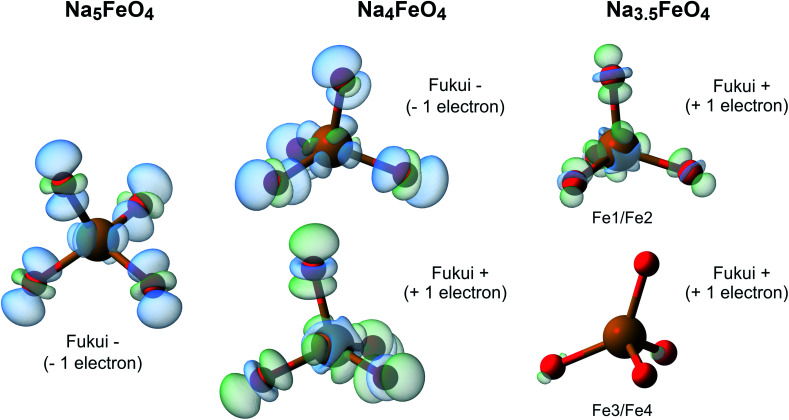
The Fukui functions computed for the phases along the convex hull. This shows the electronic reorganisation upon addition (Fukui+) and removal (Fukui−) of one electron to the system, being visible as a decrease (blue) or an increase (green) of electronic density. The isosurface value is 0.002 in all cases. Atomic labels refer to Wyckoff positions given in Table S4.† Fe1/Fe2 and Fe3/Fe4 are pairwise almost identical to each other, and are treated as two distinct Fe-sites.

To further investigate the nature of the redox in Na_5_FeO_4_, we computed the Fukui function at all desodiation levels. To compute these, we keep the structures frozen, and recompute the charge densities with either one electron added (Fukui+) or subtracted (Fukui−) from the system, revealing the spatial localisation of the lowest unoccupied and the highest occupied electronic levels respectively. A visual representation of the results from these calculations is shown in Fig. 13. This reveals that along the whole cycling interval, the electronic levels associated with the charge compensation are hybridised Fe/O-states, indicating that all redox activity is contained to these levels, and again shows that the O 2p lone pairs are left intact.

The emptying of electron states from the covalent Fe–O bonds is also predicted to occur in the Li-rich cathode material Li_1.2_Nb_0.3_Fe_0.4_O_2_ with the simultaneous formation of a superoxide species (O^−^_2_) between two oxide ions of neighbouring octahedra, with a bond length of 1.36 Å.^55^ This is not a stable situation, and leads to a reductive coupling with the oxidised Fe-cation, and leads to irreversible oxygen loss. While shortening of O–O distances is also predicted upon desodiation of Na_5_FeO_4_, these distances are still far above what could be considered superoxide or even peroxide species (O^2−^_2_), with the shortest O–O separation at *x* = 1.5 being 2.67 Å, as seen in Fig. S14.† This is more in line with what is observed in another tridimensional model system, β-Li_2_IrO_3_ (2.53 Å), that exhibits reversible electrochemical cycling without any oxygen loss.^56,57^

## 6 Conclusion

The sodium-rich oxoferrate Na_5_FeO_4_ was investigated as a potential cathode for SIBs from first principles using density functional theory. Out of multiple starting geometries, we find three low-energy orthorhombic polymorphs related to the antifluorite structure lying within 100 meV f.u.^−1^ of each other, including the experimentally reported structure that is found to be favoured at temperatures below typical synthesis temperatures upon inclusion of entropic contributions. All polymorphs are found to be both mechanically and dynamically stable. NEB calculations point toward excellent diffusive properties, although further work is needed to investigate the role of interstitial and/or interstitialcy diffusion in these materials. Investigations are made into relative phase stability as sodium is removed from the structure. We have looked into direct desodiation of the pristine structures, phase transformations and possible oxygen loss, revealing the existence of two possible reversible cycling intervals: Na_5_FeO_4_ ⇌ Na_3.5_FeO_4_ + 1.5Na^+^ and Na_3.5_FeO_3_ ⇌ Na_2_FeO_3_ + 1.5Na^+^. While the first interval shows promise with a specific energy density of more than 360 W h kg^−1^, the second interval is limited by too severe reconstructions to be a likely candidate as a cathode material. Analysis of the electronic structure, including the computation of the electron localisation function and the Fukui functions, point towards a redox mechanism contained to hybridised Fe 3d-/O 2p-levels where FeO_4_-tetrahedra serve as a joint redox centre during de-/sodiation, while unhybridised O 2p “lone pairs”, usually reported to be involved in the anionic redox process, are left untouched.

If realised experimentally, Na_5_FeO_4_ has the potential of becoming a very inexpensive cathode material for use in stationary storage applications, consisting of only cheap and geographically abundant raw materials. The results presented show that it warrants further experimental investigations, and such investigations are currently underway.

## Conflicts of interest

There are no conflicts to declare.

## Supplementary Material

RA-012-D2RA01834A-s001
