# Supplementary material for: Antifluorite-type Na5FeO4 as a low-cost, environment-friendly cathode with combined cationic/anionic redox activity for sodium ion batteries: a first-principles investigation
Source: RSC Adv. 2022 Jun 13;12(27):17410–21. doi: 10.1039/d2ra01834a (PMC9189622; doi:10.1039/d2ra01834a)
Supplement: RA-012-D2RA01834A-s001 [file RA-012-D2RA01834A-s001.pdf]

Electronic Supplementary Material (ESI) for RSC Advances.
This journal is © The Royal Society of Chemistry 2022

Electronic Supplementary Information (ESI): Antifluorite-type Na_5FeO_4 as a low-cost, environment-friendly cathode with combined cationic/anionic redox activity for sodium ion batteries: A first-principles investigation

Rasmus Vester Thøgersen, Federico Bianchini,
Helmer Fjellvåg, Ponniah Vajeeston*

[a]Centre for Materials Science and Nanotechnology, Department of Chemistry, University of Oslo,
P.O. Box 1033 Blindern, N-0315 Oslo, Norway.

*To whom correspondence should be addressed:
vajeeston.ponniah@smn.uio.no

1 Supplementary figures

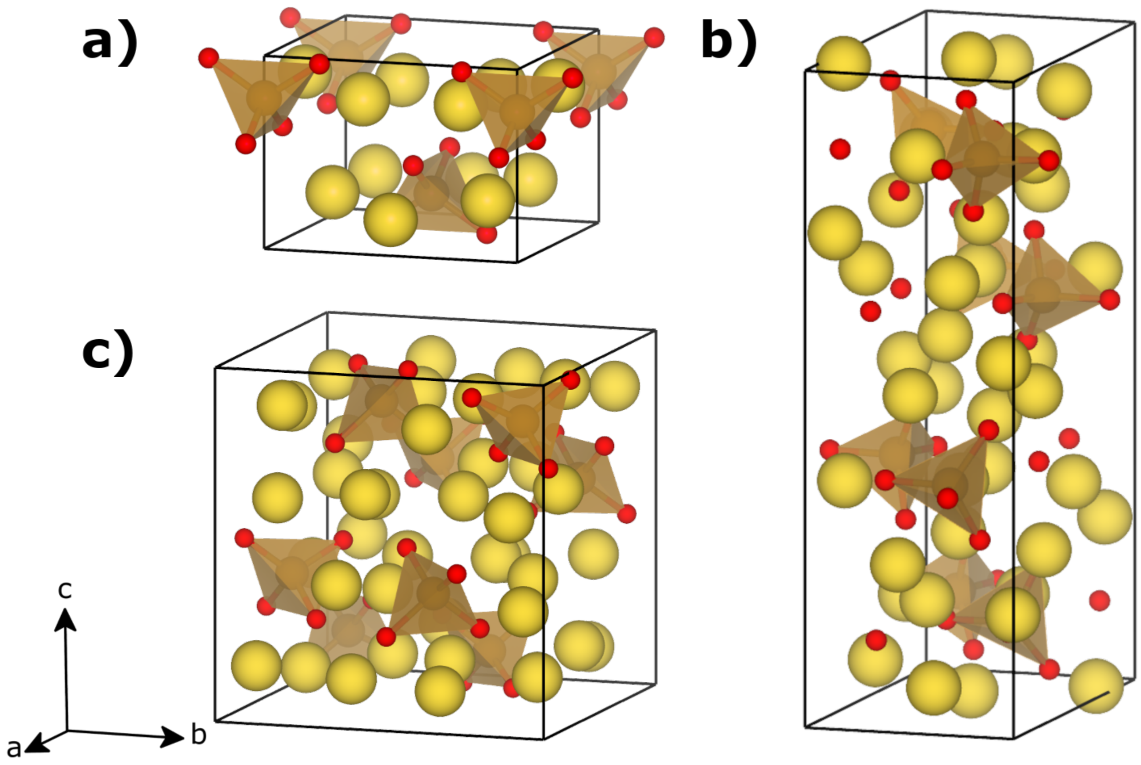

Figure S1: Primitive unit cells of the relaxed low-energy structures, where Na is yellow, Fe is brown and O is red. a) β-Na_5FeO_4 ($Pmmn$), b) α-Na_5FeO_4 ($Pbca$) and c) γ-Na_5FeO_4 ($Pbca$). Strucutral data given in Table S4.

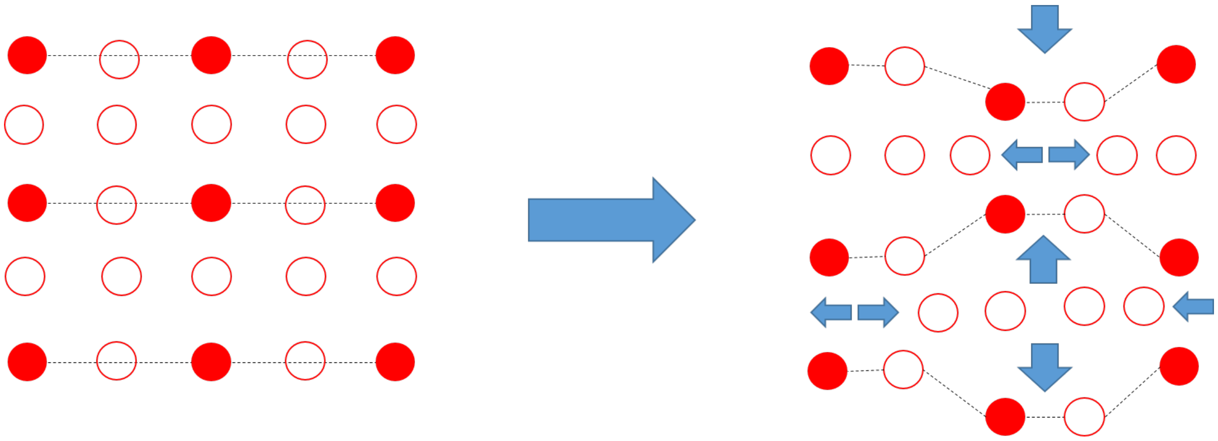

Figure S2: Schematic showing the relationship between the oxygen sublattice in the ideal anti-fluorite structure and the distorted structure of γ-Na_5FeO_4. Notice the alternating depressions and expansions for each octant.

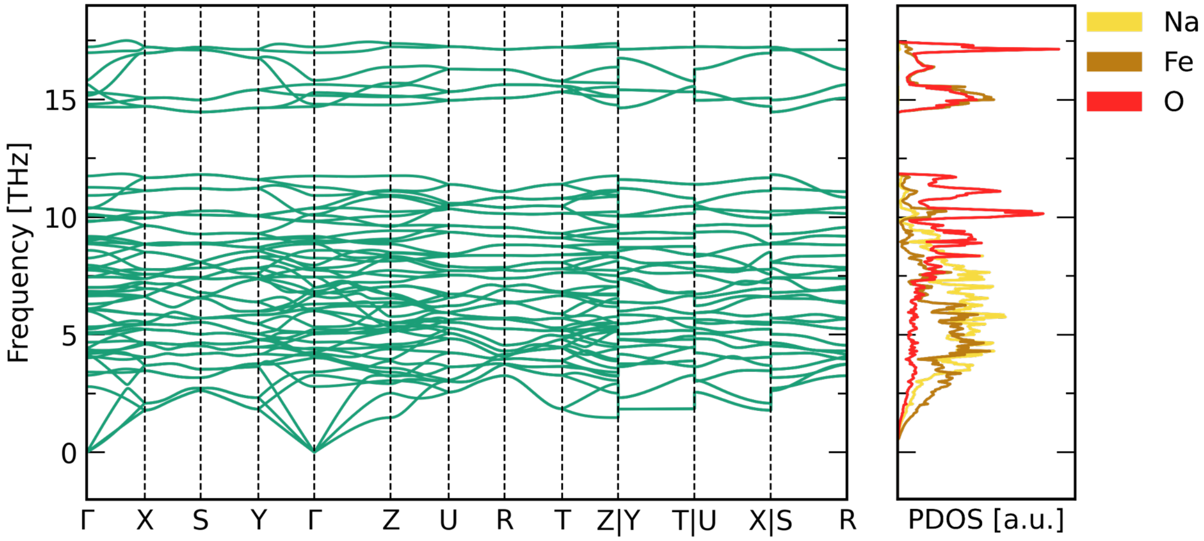

Figure S3: The phonon dispersion relation and phonon density of states for β-Na_5FeO_4.

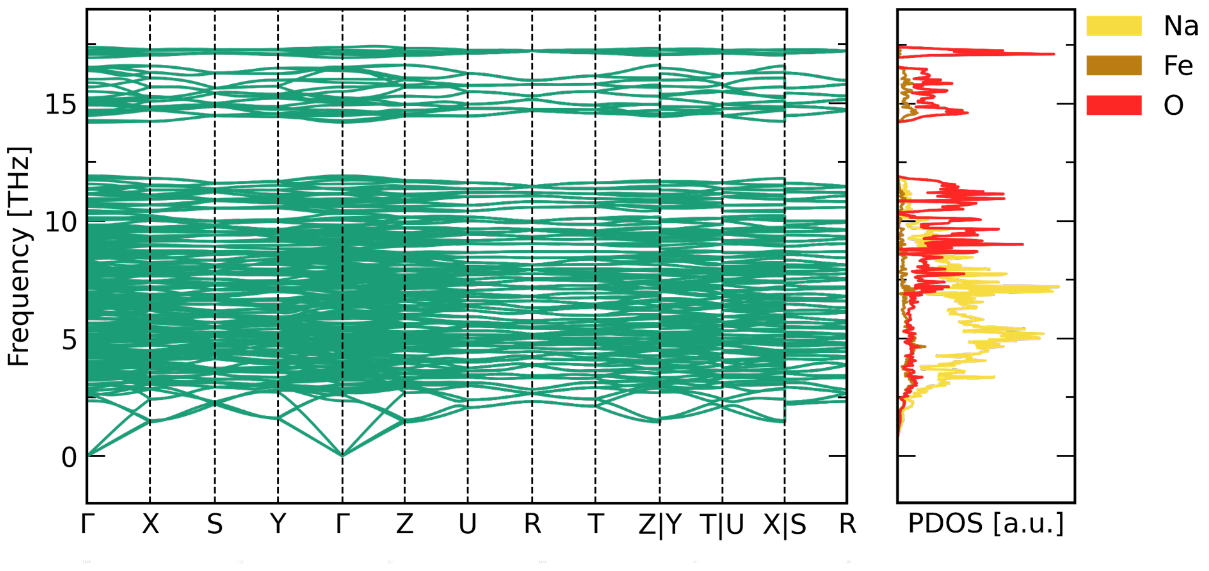

Figure S4: The phonon dispersion relation and phonon density of states for α-Na_5FeO_4.

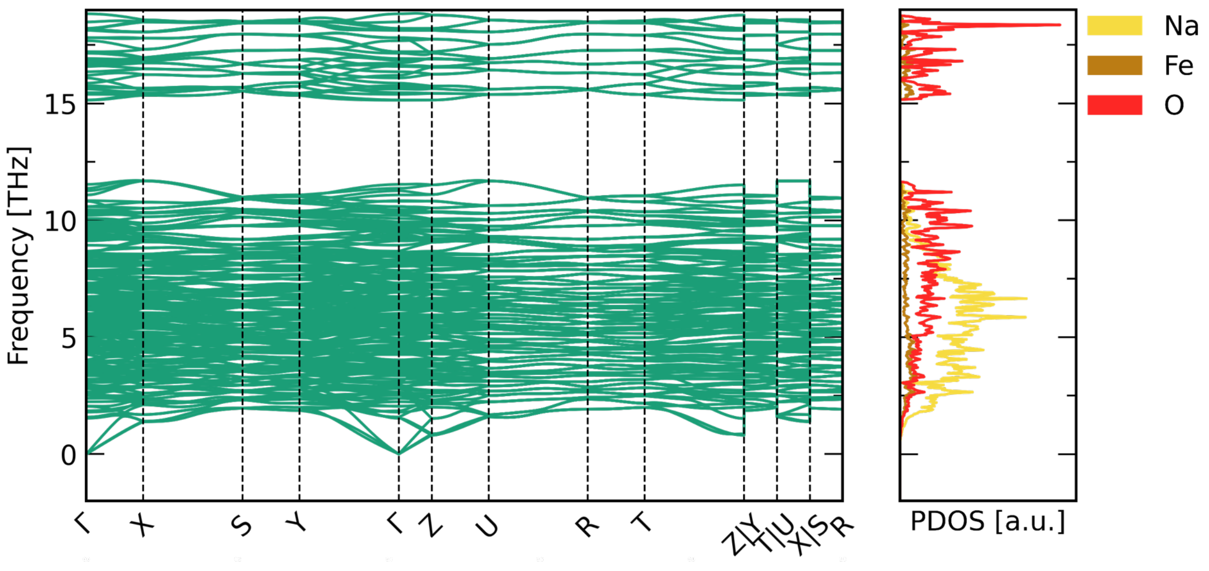

Figure S5: The phonon dispersion relation and phonon density of states for the γ-Na_5FeO_4.

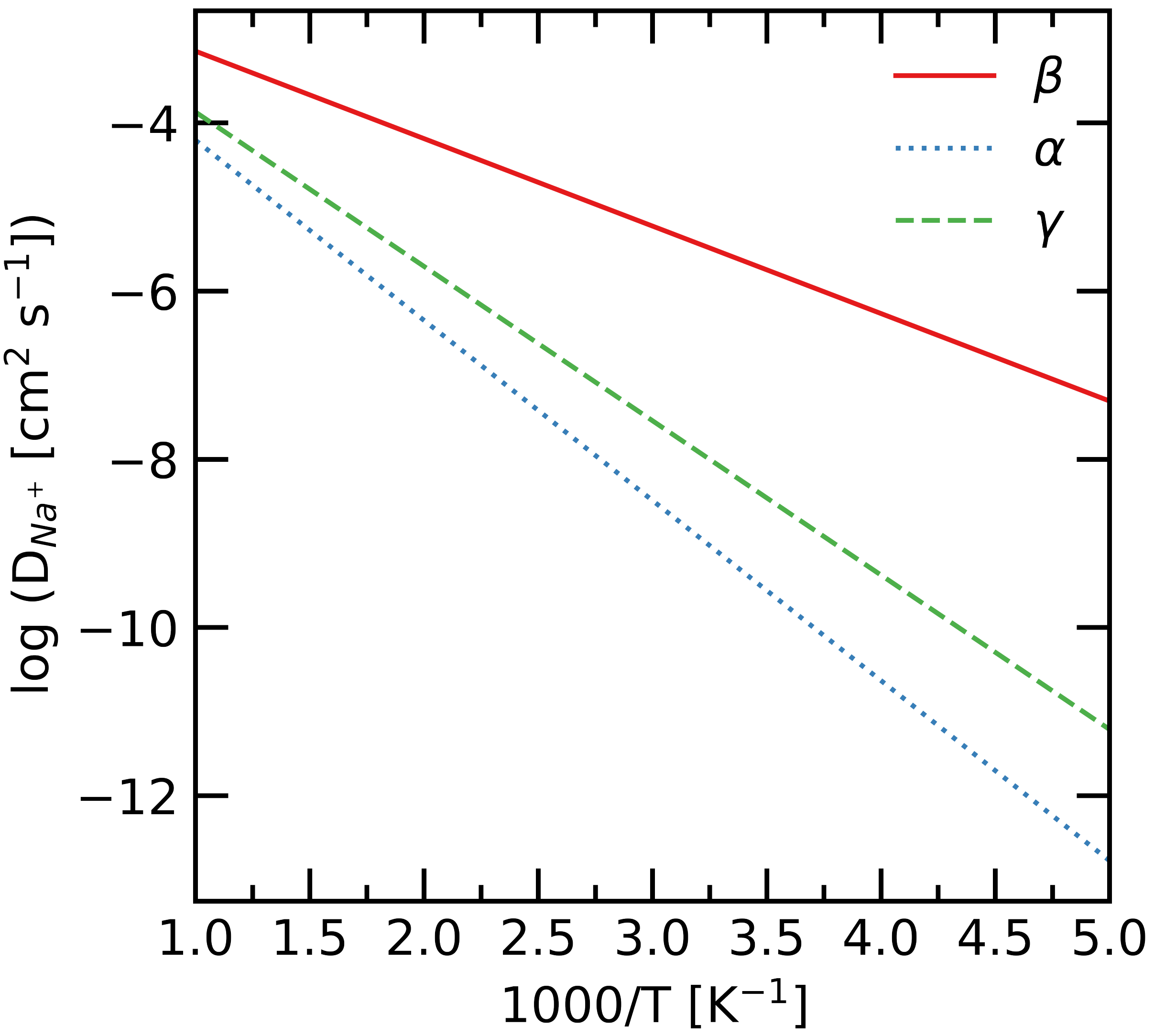

Figure S6: Arrhenius plot of the diffusion coefficients as a function of temperature based on the limiting jump along the most facile diffusion paths for each polymorph. The attempt frequency is set to 10 THz.

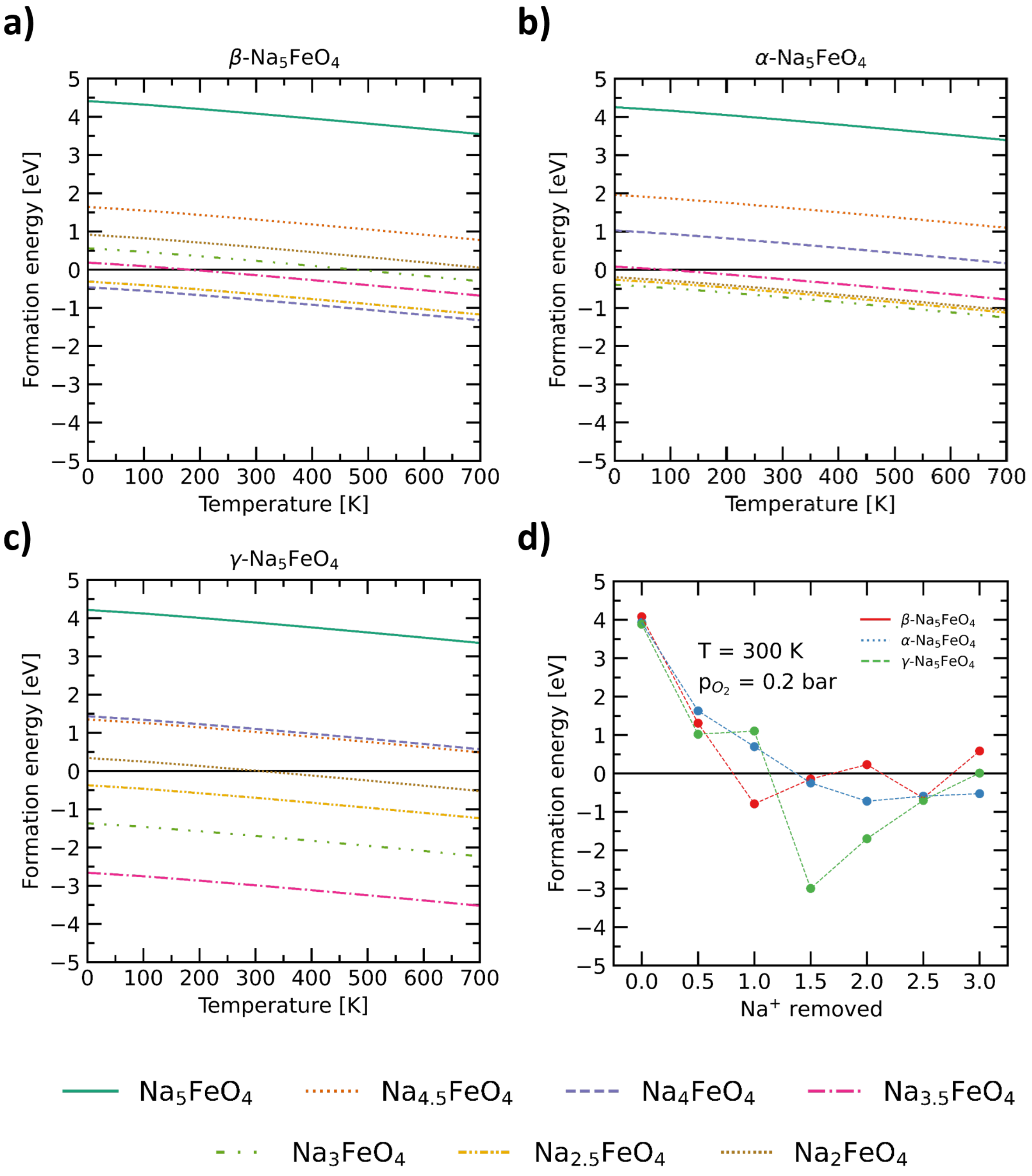

Figure S7: The evolution of oxygen vacancy formation energies as a function of temperature. Each line corresponds to a given desodiation level for each polymorph (**a-c**) for a fixed oxygen partial pressure ($p_{O_2} = 0.2$ bar), while **d)** shows the evolution of the formation energies for each polymorph as a function of desodiation at a fixed temperature (300 K) and oxygen partial pressure (0.2 bar).

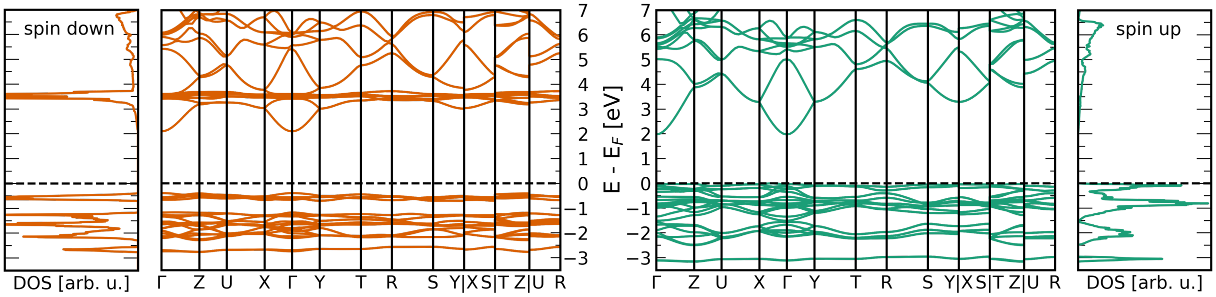

Figure S8: Total density of states and band structure along a high-symmetry path in the BZ for the β-phase, split into contributions from the spin down and spin up channels, respectively, obtained from GGA+U calculations. The Fermi-level (E_F) is by convention set to 0 and is indicated by a horizontal, dashed black line.

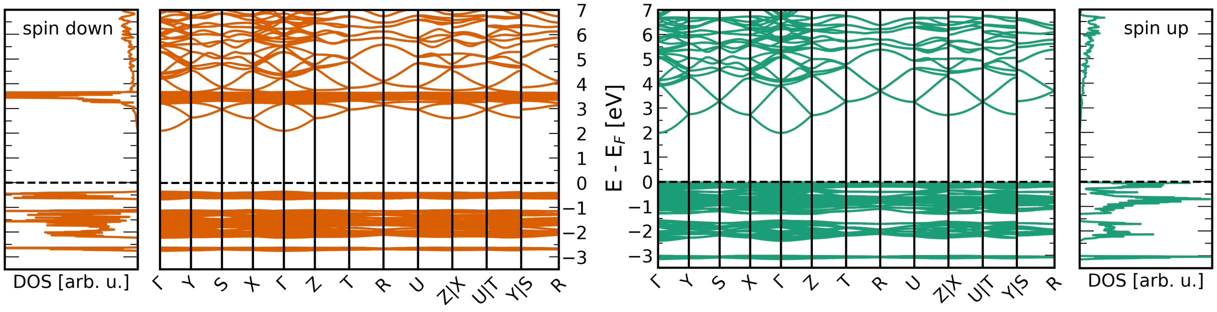

Figure S9: Total density of states and band structure along a high-symmetry path in the Brillouin zone for α-Na_5FeO_4, split into contributions from the spin down and spin up channels, respectively, obtained from GGA+U calculations. The Fermi-level is by convention set to 0 and is indicated by a horizontal, dashed black line.

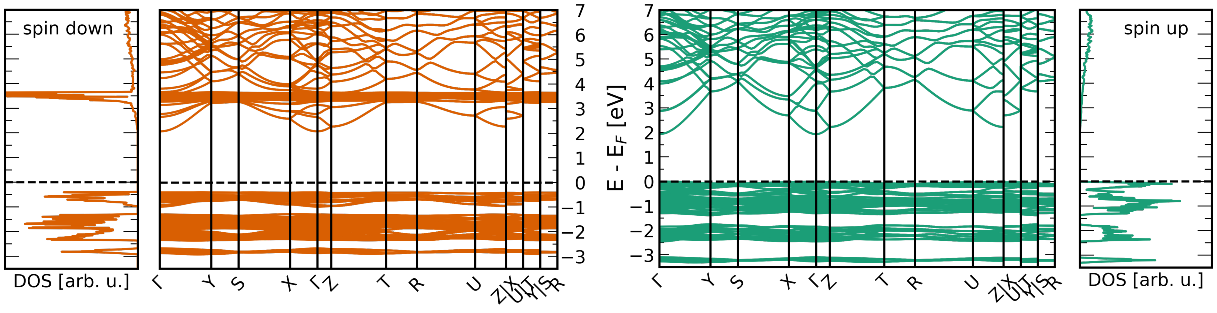

Figure S10: Total density of states and band structure along a high-symmetry path in the Brillouin zone for γ-Na_5FeO_4, split into contributions from the spin down and spin up channels, respectively, obtained from GGA+U calculations.The Fermi-level is by convention set to 0 and is indicated by a horizontal, dashed black line.

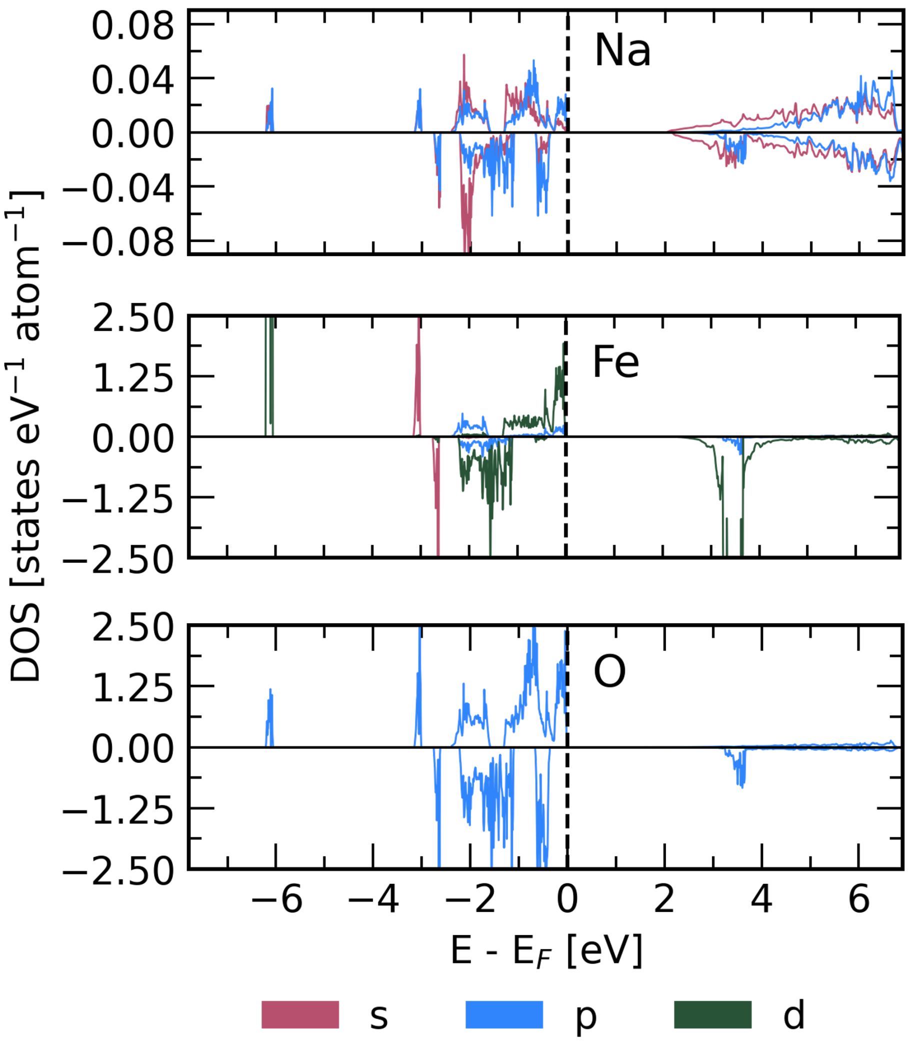

Figure S11: Site-projected electronic density of states for Na, Fe and O, respectively, for α-Na_5FeO_4 obtained from GGA+U calculations. The spin up channel is plotted with positive values and the spin down channel is plotted with negative values. The Fermi-level is by convention set to 0 and is indicated by a vertical, dashed black line.

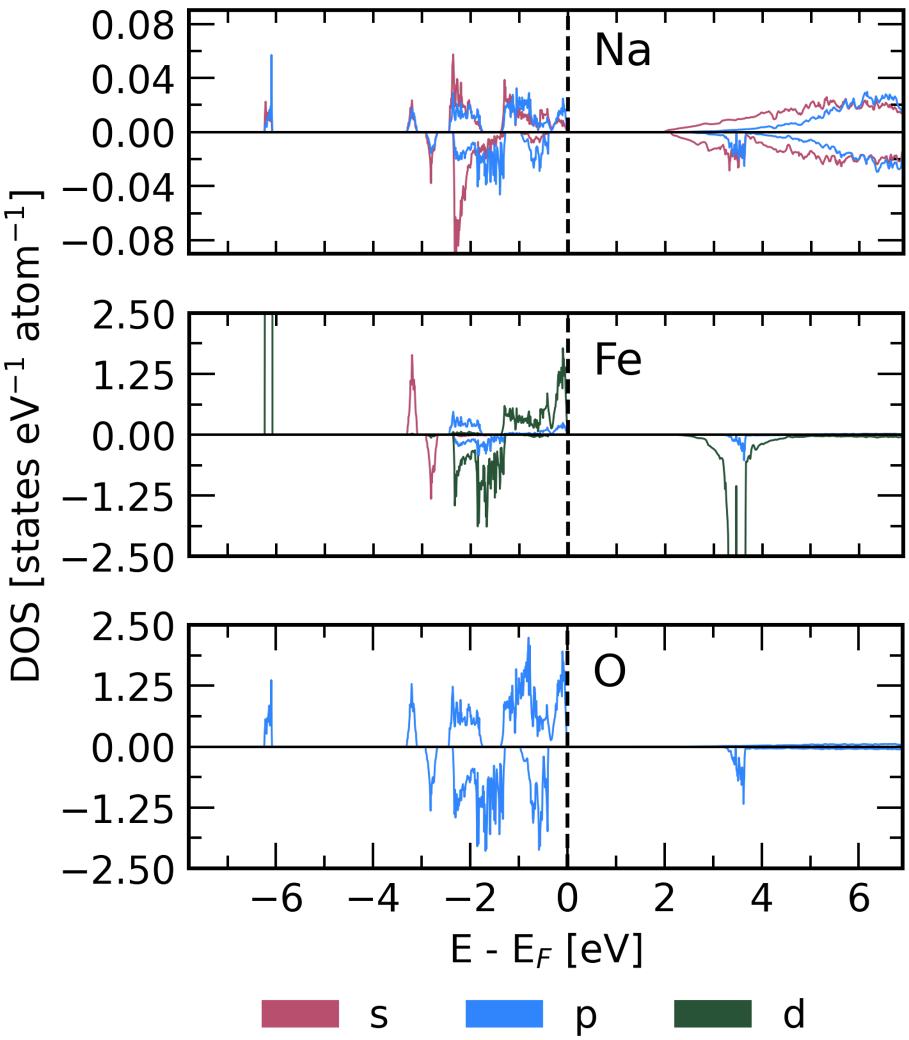

Figure S12: Site-projected electronic density of states for Na, Fe and O, respectively, for γ-Na_5FeO_4 obtained from GGA+U calculations. The spin up channel is plotted with positive values and the spin down channel is plotted with negative values. The Fermi-level is by convention set to 0 and is indicated by a vertical, dashed black line.

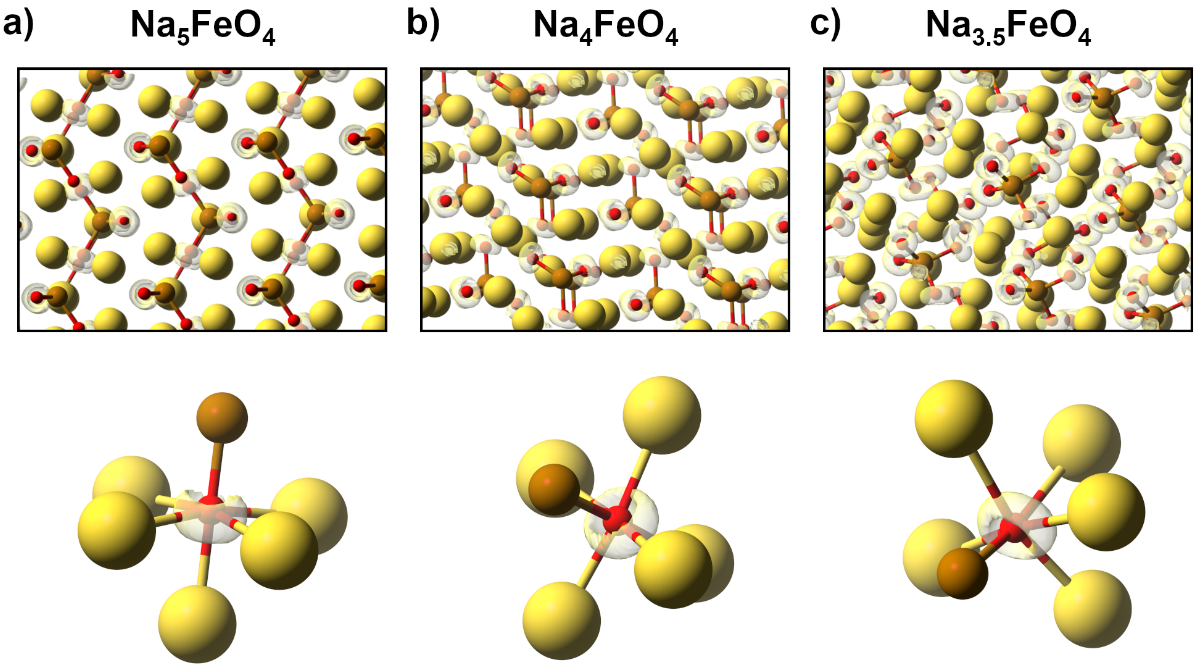

Figure S13: The electronic localisation function computed for **a)** Na_5FeO_4, **b)** Na_4FeO_4 and **c)** $Na_{3.5}FeO_4$ with an isosurface value of 0.65. A cutout of each crystal structrure is shown together with an isolated oxide ion and its nearest neighbours. There is no discernable change in the localised electron pairs surrounding the oxygen ions throughout each desodiation step, showing that these are predicted to be inactive in the redox process.

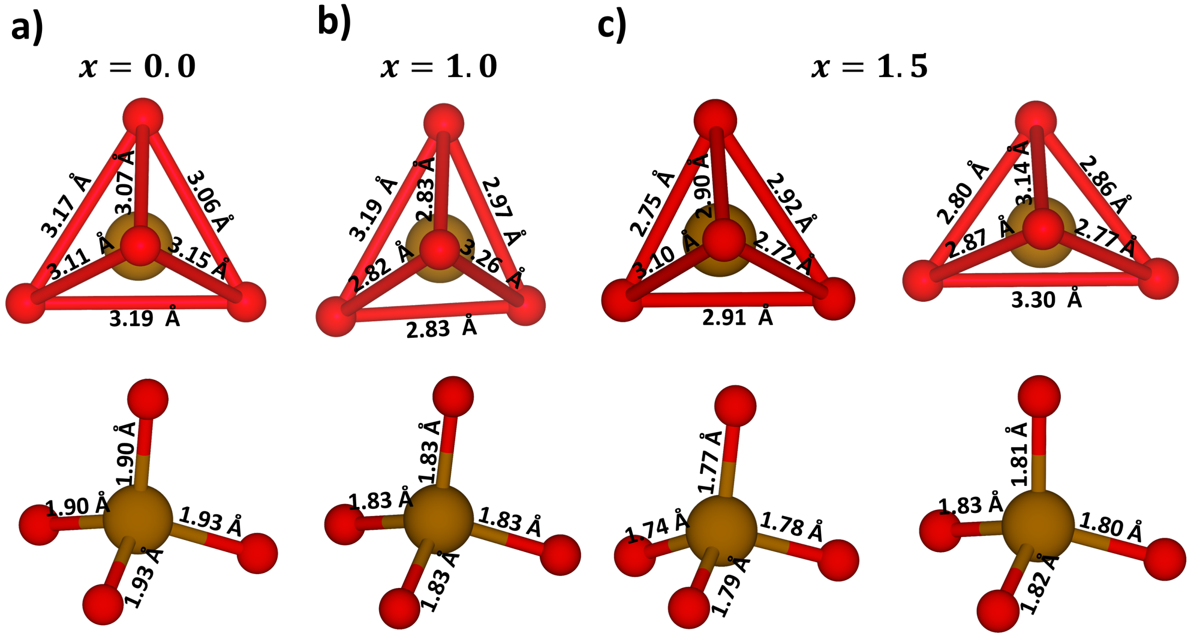

Figure S14: O-O and Fe-O distances in $Na_{5-x}FeO_4$ for **a)** $x = 0.0$, **b)** $x = 1.0$ and **c)** $x = 1.5$. There is a contraction of certain O-O distances as the compound is desodiated, as well as a shortening of the Fe-O distances, consistent with redox contributions from both Fe and O. At $x = 1.5$, there is a disproportionation between two distinct FeO_4-tetrahedra, where one is essentially unchanged from $x = 1.0$ and the other has further contraction of both O-O and Fe-O distances.

2 Supplementary tables

Table S1: List of all starting geometries and their corresponding space groups. The reference structure along with its Materials Project- (mp) or ICSD-identifier is given depending on the source.

Label	Space group	Crystal system	Reference compound	Reference
C2/m	*C2/m* (12)	Monoclinic	Li_5AuO_4	mp-1222442
$P2_1/c$ #1	$P2_1/c$ (4)		Rb_5FeO_4	mp-770083
$P2_1/c$ #2			Li_5FeO_4	mp-771402
$P2_1/c$ #3			Cs_5FeO_4	ICSD-414483
$P2_1/c$ #4			Rb_5FeO_4	mp-770716
*Aea*2	*Aea*2 (41)	Orthorhombic	Li_5CoO_4	mp-775093
Fddd	*Fddd* (70)		Li_5AuO_4	mp-757242
Pbca #1	*Pbca* (61)		Na_5FeO_4	ICSD-2485
Pbca #2			Na_5CrO_4	mp-780922
Pmmn	*Pmmn* (59)		Na_5CrO_4	mp-780916
$Pmn2_1$	$Pmn2_1$ (31)		Na_5MnO_4	ICSD-47101
Pnnm	*Pnnm* (58)		Li_5CoO_4	mp-773454
$P4_12_12$	$P4_12_12$ (92)	Tetragonal	Na_5LaO_4	mp-37207
$P4_2/nmc$	$P4_2/nmc$ (137)		Li_5GaO_4	mp-780218
*P*1	*P*1 (1)	Triclinic	Li_5FeO_4	mp-780192
$P\bar{1}$	$P\bar{1}$ (2)		K_5InO_4	ICSD-74911

Table S2: Overview of the relative equilibrium energies (ΔE) with respect to the most stable structure (β), the equilibrium volumes (V_0), the bulk moduli (B_0) and its pressure derivative (B'_0) of all starting geometries

**Structure**	ΔE **[eV f.u.$^{-1}$]**	V_0 **[Å^3 f.u.$^{-1}$]**	B_0 **[GPa]**	B'_0
Pmmn (β)	0.000	148.25	53	4.87
Pbca #2 (α)	0.027	147.36	52	5.71
Pbca #1 (γ)	0.086	144.77	47	4.33
$Pmn2_1$	0.131	142.36	53	5.44
$P2_1/c$ #1	0.160	146.05	42	6.29
$P2_1/c$ #3	0.168	148.44	49	5.84
$P2_1/c$ #2	0.283	141.64	47	5.54
*Aea*2	0.355	147.43	53	3.08
Pnnm	0.367	144.94	39	5.95
*P*1	0.402	150.22	48	4.73
$P2_1/c$ #4	0.604	148.66	39	5.37
$P4_2/nmc$	0.933	152.97	45	5.87
Fddd	1.159	145.38	53	3.83
*C*2/*m*	1.173	145.69	52	3.20
$P\bar{1}$	1.528	130.59	56	3.24
$P4_12_12$	1.900	150.31	45	10.92

Table S3: The full symmetrised elastic moduli for all three polymorphs

		**XX**	**YY**	**ZZ**	**XY**	**XZ**	**YZ**
β-Na_5FeO_4	**XX**	890.63	321.25	395.46	0.0	0.0	0.0
	**YY**	321.25	968.50	306.13	0.0	0.0	0.0
	**ZZ**	395.46	306.13	951.53	0.0	0.0	0.0
	**XY**	0.0	0.0	0.0	193.10	0.0	0.0
	**XZ**	0.0	0.0	0.0	0.0	338.76	0.0
	**YZ**	0.0	0.0	0.0	0.0	0.0	148.86
		**XX**	**YY**	**ZZ**	**XY**	**XZ**	**YZ**
α-Na_5FeO_4	**XX**	782.13	396.78	353.29	0.0	0.0	0.0
	**YY**	396.78	799.82	381.03	0.0	0.0	0.0
	**ZZ**	353.29	381.03	894.43	0.0	0.0	0.0
	**XY**	0.0	0.0	0.0	304.24	0.0	0.0
	**XZ**	0.0	0.0	0.0	0.0	315.73	0.0
	**YZ**	0.0	0.0	0.0	0.0	0.0	279.05
		**XX**	**YY**	**ZZ**	**XY**	**XZ**	**YZ**
γ-Na_5FeO_4	**XX**	704.97	278.57	326.51	0.0	0.0	0.0
	**YY**	278.57	907.28	307.65	0.0	0.0	0.0
	**ZZ**	326.511	307.65	874.496	0.0	0.0	0.0
	**XY**	0.0	0.0	0.0	275.68	0.0	0.0
	**XZ**	0.0	0.0	0.0	0.0	286.97	0.0
	**YZ**	0.0	0.0	0.0	0.0	0.0	260.57

Table S4: Structural data for the three low energy polymorphs identified in the calculations. Experimental values for the γ-polymorph (ICSD #2485) given in parenthesis

**Phase**	**Lattice parameters [Å]**			**Wyckoff site occupation**				
	$\boldsymbol{a}$	$\boldsymbol{b}$	$\boldsymbol{c}$	**Site label**	**Wyckoff site**	$\boldsymbol{x}$	$\boldsymbol{y}$	$\boldsymbol{z}$
β-Na_5FeO_4	7.228	7.472	5.473	Na1	8*g*	0.4892	0.4434	0.2575
$Pmmn$				Na2	2*b*	0.2500	0.7500	0.1865
				Fe1	2*a*	0.2500	0.2500	0.7815
				O1	4*e*	0.4739	0.4688	-0.0270
				O2	4*f*	0.4739	0.25000	0.5904
γ-Na_5FeO_4	10.523	6.023	18.196	Na1	8*c*	0.1738	0.6039	0.8286
$Pbca$	(10.334)	(5.974)	(18.082)	Na2	8*c*	0.0609	0.5827	0.4350
				Na3	8*c*	0.4221	0.6002	0.7522
				Na4	8*c*	0.3359	0.5906	0.5024
				Na5	8*c*	0.3010	0.5758	0.1408
				Fe1	8*c*	0.0400	0.6730	0.1236
				O1	8*c*	0.3643	0.7635	0.3746
				O2	8*c*	0.1257	0.7655	0.2124
				O3	8*c*	0.1327	0.6968	0.5450
				O4	8*c*	0.4522	0.6419	0.6213
α-Na_5FeO_4	10.597	10.360	10.710	Na1	8*c*	0.1067	0.1682	0.6156
$Pbca$				Na2	8*c*	0.1160	0.6437	0.4005
				Na3	8*c*	0.1204	0.6034	0.6637
				Na4	8*c*	0.1220	0.0852	0.3566
				Na5	8*c*	0.1529	0.1380	0.8840
				Fe1	8*c*	0.1466	0.6136	0.1300
				O1	8*c*	0.0250	0.7271	0.0493
				O2	8*c*	0.0512	0.5028	0.2426
				O3	8*c*	0.2420	-0.0011	0.5214
				O4	8*c*	0.2452	0.7223	0.2392

Table S5: Summary of vacancy jumps, identified by which symmetry equivalent sites the jump is between and with jump length (Å) based on the distance between the sites in the equilibrium structure, jump barriers (eV) calculated using the cNEB method and diffusion coefficients (cm^2 s^{-1}) calculated at 300 K according to equation 4. Jumps marked with asterisk are not properly described as a vacancy jump.

Phase	Jump	Label	Length	Barrier	Diffusion coefficient
β	Na1-Na1	01	2.79	0.207	2.7×10^{-6}
	Na1-Na1	02	2.89	0.036	2.1×10^{-3}
	Na1-Na2	03	2.90	0.301	7.3×10^{-8}
	Na1-Na1	04	2.95	0.405	1.4×10^{-9}
α	Na3-Na4	01	2.84	0.076	4.3×10^{-4}
	Na2-Na3	02	2.85	0.295	8.9×10^{-8}
	Na1-Na4	03	2.91	0.350	1.1×10^{-8}
	Na2-Na4	04	2.88	0.425	6.0×10^{-10}
	Na1-Na5	05	2.93	0.405	1.4×10^{-9}
	Na2-Na5	06	2.95	0.373	4.7×10^{-9}
	Na4-Na5	07	2.90	0.470	1.1×10^{-10}
	Na1-Na3	08	3.01	0.360	8.0×10^{-9}
	Na3-Na5	09	2.96	0.443	3.1×10^{-10}
	Na1-Na2*	10	3.07	0.071	6.0×10^{-4}
γ	Na2-Na2	01	2.87	0.120	8.0×10^{-5}
	Na1-Na2	02	2.95	0.072	5.5×10^{-4}
	Na1-Na3*	03	2.96	0.035	2.2×10^{-3}
	Na1-Na3	04	3.03	0.364	7.0×10^{-9}

Table S6: Summary of the percolating diffusion paths of jumps between Na-sites, where the distance between each Na^{+}-ion is less than 3 Å, as calculated using the cNEB-method. The path is described by which symmetry equivalent Na^{+}-ions, as detailed in Table S4, are involved in each path. For each diffusion path, the direction of the net movement, the overall activation barrier (E_a) associated with the energy path and the diffusion coefficient at 300 K (D_{Na^+}) as calculated from Equation 4 using the limiting jump along the path. Diffusion paths marked with an asterisk (*) include jumps which are not properly described as a vacancy jump, and values are for this reason more uncertain.

**Phase**	**No.**	**Path**	**Direction**	$\boldsymbol{E_a}$ [eV]	$\boldsymbol{D_{Na^+}(T = 300\ K)}$ [$cm^2\ s^{-1}$]
β-Na_5FeO_4	1	Na1 → Na1 → Na1	[110]	0.206	2.7×10^{-6}
	2	Na1 → Na2 → Na1 → Na1	[1$\bar{1}$0]	0.301	7.3×10^{-8}
	4	Na1 → Na2 → Na1 →Na1	[1$\bar{1}$0]	0.405	1.4×10^{-9}
	3	Na1 → Na1 → Na1	[110]	0.406	1.4×10^{-9}
	5	Na1 → Na1 → Na1	[001]	0.406	1.4×10^{-9}
α-Na_5FeO_4	1	Na3 → Na4 → Na2 → Na3	[001]	0.434	6.0×10^{-10}
	2	Na5 → Na4 → Na2 → Na5	[010]	0.470	1.1×10^{-10}
	3	Na3 → Na4 → Na1 → Na5 → Na2 → Na3	[001]	0.478	1.1×10^{-10}
	4*	Na1 → Na3 → Na4 → Na2 → Na2 → Na1	[100]	≥ 0.521	$\leq 1.1 \times 10^{-10}$
γ-Na_5FeO_4	1*	Na1 → Na3 → Na1	[100]	≥ 0.387	$\leq 7.0 \times 10^{-9}$
	2*	Na1 → Na3 → Na1 → Na2 → Na2 → Na1	[001]	≥ 0.424	$\leq 7.0 \times 10^{-9}$

Table S7: Structural data for the phases found along the convex hull in the cycling interval $Na_5FeO_4 \rightleftharpoons Na_{3.5}FeO_4 + 1.5\,Na^+$. The pristine phase, Na_5FeO_4 is not given here and can be found in Table S4.

**Phase**	**Lattice parameters [Å]**			**Wyckoff site occupation**				
	$\boldsymbol{a}$	$\boldsymbol{b}$	$\boldsymbol{c}$	**Site label**	**Wyckoff site**	$\boldsymbol{x}$	$\boldsymbol{y}$	$\boldsymbol{z}$
Na_4FeO_4	5.817	5.826	8.374	Na1	2*i*	0.0494	0.7242	0.4321
$P\bar{1}$	α = 87.923			Na2	2*i*	0.2099	0.7458	0.0193
	β = 72.036			Na3	2*i*	0.2394	0.2369	0.1697
	γ = 69.133			Na4	2*i*	0.4146	0.7849	0.6275
				Fe1	2*i*	0.4040	0.2834	0.7504
				O1	2*i*	0.5618	0.4784	0.8019
				O2	2*i*	0.3144	-0.0310	0.3820
				O3	2*i*	0.8297	0.4984	0.3393
				O4	2*i*	0.7640	0.8875	0.1003
$Na_{3.5}FeO_4$	9.561	9.090	11.159	Na1	2*a*	0.5282	0.6410	0.1241
$P2_1$	β = 95.854			Na2	2*a*	0.4721	0.3728	0.3754
				Na3	2*a*	-0.0673	0.7083	0.8825
				Na4	2*a*	0.0688	0.3054	0.6183
				Na5	2*a*	0.2176	0.1768	0.3667
				Na6	2*a*	0.8107	0.0616	0.8968
				Na7	2*a*	0.4104	-0.0273	0.1228
				Na8	2*a*	0.5885	0.0407	0.3771
				Na9	2*a*	0.2175	0.3389	0.8673
				Na10	2*a*	0.8111	0.4536	0.3965
				Na11	2*a*	0.1528	0.5177	0.3779
				Na12	2*a*	0.8479	0.4978	0.1214
				Na13	2*a*	0.2949	0.6659	0.8719
				Na14	2*a*	0.7069	0.3483	0.6285
				Fe1	2*a*	0.1083	0.8647	0.1272
				Fe2	2*a*	0.8920	0.1504	0.3731
				Fe3	2*a*	0.3744	0.2083	0.6176
				Fe4	2*a*	0.6263	0.8054	0.8827
				O1	2*a*	0.0690	0.7196	0.2269
				O2	2*a*	-0.0664	0.2956	0.2748
				O3	2*a*	0.5059	0.3059	0.7138
				O4	2*a*	0.4958	0.7065	0.7864
				O5	2*a*	-0.0212	-0.0568	0.0209
				O6	2*a*	0.0208	0.0735	0.4803
				O7	2*a*	0.3893	0.0511	0.5173
				O8	2*a*	0.6100	-0.0383	-0.0163
				O9	2*a*	0.1817	0.5178	0.7267
				O10	2*a*	0.8182	0.4984	0.7734
				O11	2*a*	0.2681	0.3788	0.2303
				O12	2*a*	0.7326	0.6373	0.2693
				O13	2*a*	0.2295	0.7989	0.0278
				O14	2*a*	0.7697	0.2150	0.4710
				O15	2*a*	0.3008	0.3445	0.5107
				O16	2*a*	0.7002	0.6691	-0.0107

Table S8: Structural data for the phases found along the convex hull in the cycling interval $Na_{3.5}FeO_3 \rightleftharpoons Na_2FeO_3 + 1.5\,Na^+$.

**Phase**	**Lattice parameters [Å]**			**Wyckoff site occupation**				
	a	b	c	**Site label**	**Wyckoff site**	x	y	z
$Na_{3.5}FeO_3$	6.027	6.407	6.276	Na1	1*a*	0.9907	0.3781	0.2207
$P1$	α = 120.488			Na2	1*a*	0.7806	0.0002	0.8254
	β = 91.058			Na3	1*a*	0.6611	0.6998	0.1807
	γ = 88.168			Na4	1*a*	0.3646	0.3112	0.8779
				Na5	1*a*	0.3503	-0.0100	0.1333
				Na6	1*a*	0.0314	0.6263	0.8399
				Na7	1*a*	0.0271	0.0592	0.4870
				Fe1	1*a*	0.3283	0.7086	0.5166
				Fe2	1*a*	0.6171	0.3519	0.5171
				O1	1*a*	0.7355	0.0746	0.2271
				O2	1*a*	0.7413	0.3569	0.8028
				O3	1*a*	0.6841	0.6360	0.5060
				O4	1*a*	0.2918	0.3621	0.5142
				O5	1*a*	0.2362	0.6799	0.2016
				O6	1*a*	0.1782	0.9721	0.8044
Na_3FeO_3	5.860	12.750	6.268	Na1	4*e*	0.0502	0.2221	0.1953
$P2_1/c$	β = 121.849			Na2	4*e*	0.0360	0.4845	0.2807
				Na3	4*e*	0.5740	0.5946	0.8588
				Fe1	4*e*	0.5772	0.3357	0.7564
				O1	4*e*	-0.0588	0.3733	-0.0508
				O2	4*e*	0.6806	-0.0617	0.8019
				O3	4*e*	0.4776	0.7284	0.5497
Na_2FeO_3	5.376	9.302	11.060	Na1	8*f*	0.7619	0.0784	0.5003
$C2/c$	β = 99.575			Na2	4*d*	0.2500	0.2500	0.5000
				Na3	4*e*	0.0000	0.0866	0.2500
				Fe1	4*e*	0.0000	0.7545	0.2500
				Fe2	4*e*	0.0000	0.4206	0.2500
				O1	8*f*	0.8493	0.1023	0.8538
				O2	8*f*	0.5857	0.0837	0.1491
				O3	8*f*	0.6508	0.2224	0.6494

Table S9: Summary of vacancy formation enthalpies (in eV) and vacancy concentrations (% of total oxygen in the unit cell removed) for each structure at varying levels of desodiation (x in $Na_{5-x}FeO_4$).

Label	Desodiation level	Stoichiometry	Formation enthalpy	Vacancy concentration
β-NFO	0.0	$Na_5FeO_{3.9375}$	4.41	1.56 %
	0.5	$Na_{4.5}FeO_{3.9375}$	1.64	1.56 %
	1.0	$Na_4FeO_{3.9375}$	-0.46	1.56 %
	1.5	$Na_{3.5}FeO_{3.9375}$	0.18	1.56 %
	2.0	$Na_3FeO_{3.9375}$	0.56	1.56 %
	2.5	$Na_{2.5}FeO_{3.9375}$	-0.31	1.56 %
	3.0	$Na_2FeO_{3.9375}$	0.91	1.56 %
α-NFO	0.0	$Na_5FeO_{3.875}$	4.26	3.13 %
	0.5	$Na_{4.5}FeO_{3.875}$	1.96	3.13 %
	1.0	$Na_4FeO_{3.9375}$	1.03	1.56 %
	1.5	$Na_{3.5}FeO_{3.875}$	0.08	3.13 %
	2.0	$Na_3FeO_{3.875}$	-0.39	3.13 %
	2.5	$Na_{2.5}FeO_{3.875}$	-0.26	3.13 %
	3.0	$Na_2FeO_{3.9375}$	-0.20	1.56 %
γ-NFO	0.0	$Na_5FeO_{3.9375}$	4.21	1.56 %
	0.5	$Na_{4.5}FeO_{3.9375}$	1.35	1.56 %
	1.0	$Na_4FeO_{3.9375}$	1.43	1.56 %
	1.5	$Na_{3.5}FeO_{3.9375}$	-2.66	1.56 %
	2.0	$Na_3FeO_{3.9375}$	-1.37	1.56 %
	2.5	$Na_{2.5}FeO_{3.9375}$	-0.37	1.56 %
	3.0	$Na_2FeO_{3.9375}$	0.34	1.56 %
Convex hull #1	0.0	$Na_5FeO_{3.875}$	4.41	3.13 %
	1.0	$Na_4FeO_{3.875}$	1.31	3.13 %
	1.5	$Na_{3.5}FeO_{3.875}$	0.05	3.13 %
Convex hull #2	1.5	$Na_{3.5}FeO_{2.9375}$	4.19	2.08 %
	2.0	$Na_3FeO_{2.9375}$	3.43	2.08 %
	3.0	$Na_2FeO_{2.9688}$	0.46	1.04 %

Table S10: Electronic band gaps (in eV) for all polymorphs obtained from GGA+U calculations. In all cases the total band gap comes from states in the spin up channel. All band gaps are direct, but in the valence band there are states close to E_F along the full path in BZ.

**Phase**	$\mathbf{E}_{g,TOT}$	$\mathbf{E}_{g,UP}$	$\mathbf{E}_{g,DOWN}$	**Kind**
β	1.98	1.98	2.49	Direct
α	1.94	1.94	2.47	Direct
γ	1.99	1.99	2.46	Direct
